# A Comparative Study of Biomimetic Synthesis of EDOT-Pyrrole and EDOT-Aniline Copolymers by Peroxidase-like Catalysts: Towards Tunable Semiconductive Organic Materials

**DOI:** 10.3389/fchem.2022.915264

**Published:** 2022-06-29

**Authors:** Manuel Eduardo Martínez-Cartagena, Juan Bernal-Martínez, Arnulfo Banda-Villanueva, Ilse Magaña, Teresa Córdova, Antonio Ledezma-Pérez, Salvador Fernández-Tavizón, Ramón Díaz de León

**Affiliations:** ^1^ Advanced Materials Department, Research Center in Applied Chemistry (CIQA), Saltillo, México; ^2^ Laboratory in Biomedicine and Nanotechnology, Aguascalientes, México; ^3^ Polymerization Processes Department, Research Center in Applied Chemistry (CIQA), Saltillo, México

**Keywords:** Polyaniline, Polypyrrole, poly (3,4ethylenedioxythiophene), conjugated semiconductive copolymer, Biomimetic

## Abstract

It has been two decades since biomimetic synthesis of conducting polymers were first reported, however, the systematic investigation of how catalysts influence the properties of the conducting polymers has not been reported yet. In this paper, we report a comparative study between peroxidase-like catalyst, dopants, and their effect on the properties of poly (3,4-ethylenedioxythiophene) (PEDOT), polypyrrole (PPY), and polyaniline (PANI). We also investigate the EDOT-Pyrrole and EDOT-Aniline copolymerization by enzymomimetic synthesis using two catalysts (Ferrocene and Hematin). It was found that, chemically, there are no detectable effects, only having small contributions in molar ratios greater than 0.7–0.3. Spectroscopic data provide solid evidence concerning the effect in the variation of the molar fractions, finding that, as the molar fraction of EDOT decreases, changes associated with loss of the conjugation of the structure and the oxidation state of the chains were observed. The electrical conductivity was considerably modified depending on the type of catalyst. Hematin produces conductive homopolymers and copolymers when doped with *p*-toluene sulfonic acid (TSA), while ferrocene produces low conductive copolymers under the same conditions. The mole fraction affects conductivity significantly, showing that as the EDOT fraction decreases, the conductivity drops drastically for both EDOT-PY and EDOT-ANI copolymers. The type of dopant also notably affects conductivity; the best values were obtained by doping with TSA, while the lowest were obtained when doping with polystyrene sulfonate (PSS). We also draw a biomimetic route to tailor the fundamental properties of conducting homopolymers and copolymers for their design and scaled-up production, as they have recently been found to have use in a broad range of applications.

## Introduction

In recent decades, enzymes have shown viability in a wide range of industrial applications because of their unique catalytic characteristics. However, these biomolecules exhibit some limitations, for instance their challenging extraction and purification implies a high economical cost (Beilen and Li, 2002). One of the ways to overcome this limitation consists of the use of enzymes as high technology catalysts, like biosensors, degradation of environmental waste, or synthesis of materials (Loos, 2010; Kucherenko et al., 2019). In the last two decades, enzymes have been demonstrated to be useful in the materials field to promote the enzymatic polymerization of several classes of polymers such as polyesters or semiconducting conjugated polymers (Parravano, 1951; Cruz-Silva et al., 2010; Romero-García et al., 2019), which are macromolecules with highly alternating sigma and π-bonds. The continuous overlapping of π-orbitals generates high electronic conjugation, leading to the formation of so-called “molecular wires”. This specific molecular architecture provides a semiconductor band structure. Additionally, these polymers possess high ion mobility on their surface. Inspired by enzymatic polymerization, several research groups have developed biomimetic synthesis. This synthesis uses small molecules or nanoparticles that resemble the active site of oxidoreductases, allowing the polymerization of different kinds of materials for various applications (Loos, 2010).

The synthesis of conjugated semiconductor polymers (CSP) through biomimetic catalysis has generated great interest in terms of its feasibility to generate new structures using low environmental impact chemicals and reusable catalysts. Poly (3,4-ethylenedioxythiophene) (PEDOT), polypyrrole (PPY), and polyaniline (PANI) precursor monomers are accessible and inexpensive, which could allow their mass production for their application in the energy, bioengineering, and electronic industries (Namsheer and Rout, 2021). However, the biomimetic synthesis of CSP made of PANI, PEDOT, and PPY remains to be fully unveiled. Solanki et *al.* electropolymerized PANI-*co*-PPY and subsequently immobilized cholesterol oxidase in the polymer to be used as a biosensor with sensitivity of 93.3 mA/mM, higher than their homopolymers Solanki et al. (2007). Controversially, the enzymomimetic synthesis PANI-*co*-PPY has been reported to generate polymers with lower electrical conductivity than their homopolymers (Lim et al., 2001; Stejskal et al., 2004; Moon et al., 2007; Mavundla et al., 2010; Astratine et al., 2014). Low conductivity, however, has been associated with the lack of optimization in the copolymer molar composition and morphological arrangement. Improved electrical conductivity in PANI-*co*-PPY has been achieved by incorporating Fe_3_O_4_ nanospheres into the CSP structure (Wang W. et al., 2016), through chemical oxidative polymerization using polystyrene sulfonate (PSS) as dopant (Huang et al., 2014), and by electro polymerization on glassy carbon (El-Enany et al., 2010) or on an indium oxide (ITO) electrode (Authidevi et al., 2016), among others. The electrochemical synthesis of PANI-co-PEDOT has also resulted in CSP with excellent electrical properties (Randriamahazaka et al., 2005; Kang et al., 2009; Kulandaivalu et al., 2015, 2016). Hematin, hemoglobin, and porphyrin are among the most relevant biomimetic catalysts recently reported (Cruz-Silva et al., 2010). Roy et *al.* reported that hematin was functionalized with poly (ethylene glycol) (PEG) chains to solubilize the molecule in an acidic aqueous medium. Hematin was subsequently used to catalyze the polymerization of aniline in the presence of lignin sulfonic acid. Hematin-PEG showed catalytic activity in the pH range of 1-4, yielding to an emeraldine-type polymer Roy et al. (2002). Nabid et *al.* used tetra (*p*-sulfonatophenyl) porphyrin as a biomimetic catalyst, showing optimal catalytic activity in a low pH range (Nabid et al., 2006). Wang et *al.* demonstrated the use of hemoglobin as a biomimetic catalyst in the synthesis of PANI-PSS. The reaction was carried out in a pH range between 1 and 4. The spectroscopic characteristics were, like the PANI, obtained enzymatically, however, the electrical conductivity decreased Wang Q. et al. (2018). Our group has also contributed to the biomimetic synthesis field using catalyst supports (Tierrablanca et al., 2010). We have also reported the fabrication of ECG electrodes by the electrophoretic deposition of EDOT-Pyrrole copolymer biomimetically synthesized using hematin (Martínez-Cartagena et al., 2021). More recently, Wang et *al.* reported the biomimetic synthesis of PANI using ferrocene, a sandwich-type metallocene widely used in organometallic chemistry. To our knowledge, this investigation was the first reporting ferrocene-based enzymomimetic polymerization, leading to a new pathway for the synthesis of CSP Wang Q. et al. (2018).

In this study, homopolymers of PEDOT, PPY, and their copolymers were synthesized for the first-time using ferrocene. We compared the polymerization of EDOT-PY and EDOT-ANI copolymers using ferrocene and hematin to determine the catalyst’s effect. The differences in conductivity, z-potential, and particle size caused by the type of peroxidase catalyst were studied, as well as the concomitant effect dependent on doping with PSS or *p*-toluene sulfonic acid (TSA). The work reported here is integrative evidence of rational modulation in CSP properties based on the type of biomimetic catalyst and dopant. Our work demonstrates biomimetic synthesis as a suitable approach for the design of application-based conductive polymers.

## Materials and Methods

### Biomimetic Synthesis of Homopolymers

**SCHEME 1:**
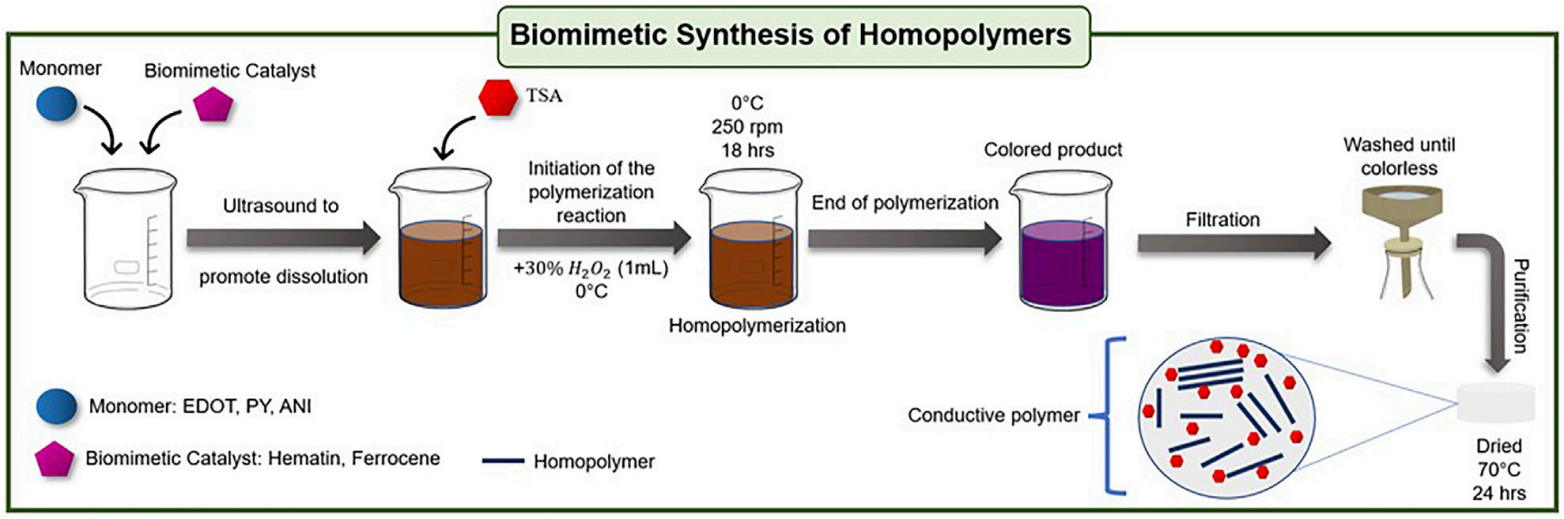
General diagram of biomimetic synthesis of homopolymers.

Homopolymers were synthesized by dissolving 10 mg of ferrocene or 10 mg of a DMSO-Hematin solution (100 mg/ml) in 200 mg of monomer (aniline, pyrrole or EDOT from Sigma Aldrich, Supplementary Table S1). The solution process was assisted by ultrasound (40 Hz) for 15 min. Then, 20 ml of a *p*-toluene sulfonic acid (Sigma Aldrich) 1M solution (pH 1.8) was added to the mixture, which was magnetically stirred for 2 h at 1000 rpm. The polymerization was initiated by the micro-dosing of 1 ml of 30% H_2_O_2_ (Fermont) using a peristaltic pump for 15 min at 0°C. Once the addition was completed, the temperature and stirring conditions (0°C, 250 rpm) were sustained for 18 h. The color of the solution changed gradually depending on the monomer: for aniline from brown to dark purple, for pyrrole from light brown to dark brown or black, and for EDOT from light yellow to dark blue. The recovered solid was filtered (0.25 µm PTFE membrane) and washed with deionized water, methanol, and acetone until the filtrate was colorless. The filtered product was dispersed in 20 ml of a binary solution of acetone-DMSO 1:1 under ultrasound for 15 min. Then, it was filtered and washed with acetone. The filtration cycle described above was repeated two more times to remove monomer residuals and low molecular weight oligomer. Finally, the product was dried at 70°C for 24 h, isolated from light, and stored for subsequent tests. Blanks were run for each monomer following the reaction protocols described above without the addition of catalyst. Scheme 1, shows an illustrative diagram of the biomimetic synthesis of homopolymers.

### Biomimetic Synthesis of Copolymers

**SCHEME 2:**
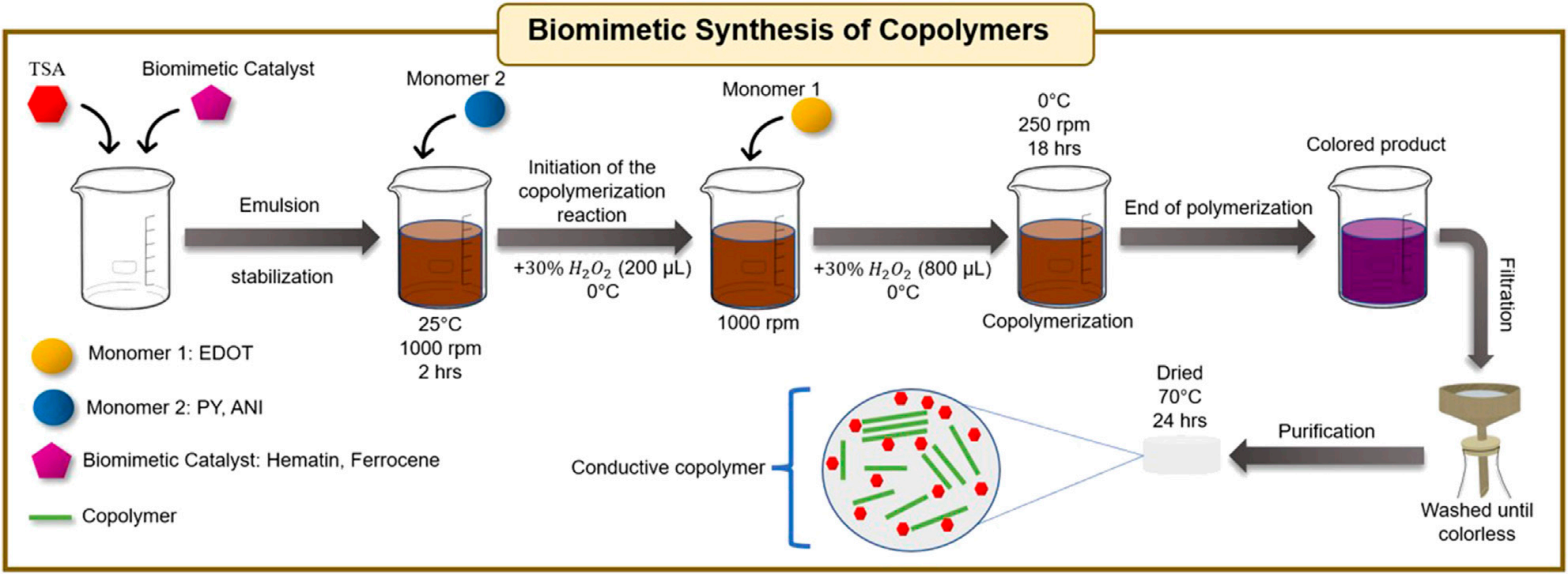
General diagram of biomimetic synthesis of copolymers.

A typical reaction requires the dissolution of 10 mg of catalyst (Ferrocene or Hematin) according to the method described above. First, 20 ml of a 1M *p*-toluene sulfonic acid solution were added and the emulsion was stabilized for 2 h under magnetic stirring. The amount of monomer used is described in Supplementary Table S2 (supplementary information Monomer 2 was added first and constant stirring was maintained at 1000 rpm for 2 h at room temperature. Polymerization was started by microdosing of 200 µl of 30% H_2_O_2_ for 30 s, keeping the system at 0°C. Then, monomer 1 was added to the system (stirring 1000 rpm), and immediately 800 µl of 30% H_2_O_2_ was micro-dosed for 20 min at 0°C. Once the addition was completed, the temperature and stirring conditions were maintained (0°C, 250 rpm) for 18 h. The copolymer was filtered on a 0.25 µm PTFE membrane and washed with deionized water, methanol, and acetone until the filtrate was colorless. Then the filtered emulsion was redispersed in 20 ml of a 1:1 acetone-DMSO binary solution under ultrasound for 15 min. It was filtered again and washed with acetone on multiple times before it was dried at 70°C for 24 h and stored in the dark for further testing. Supplementary Table S2 contains the information regarding the amounts of each reagent used, as well as the variation in % of monomers and general reaction conditions. In all cases, blanks were run following the reaction protocols described above without the addition of the catalyst (Ferrocene or Hematin). Scheme 2, shows an illustrative scheme of the biomimetic synthesis of copolymers.

### RAFT Polymerization of PSS

First, 9.7 mmol of sodium *p*-styrene sulfonate monomer, 0.0286 mmol of S-(thiobenzoyl) thioglycolic acid (TBTGA) as CTA agent, 0.0071 mmol of 4,4′-Azobis (acid 4-cyanovaleric) as initiator, and 13 ml of a 1:12 aqueous methanol: water solution was added. The solution was placed in a round-bottom flask with a magnetic stirrer sealed with a rubber septum. The solution had an initial pH of 8 and was brought to pH 1 by dropping 1N H_2_SO_4_. The reactor was purged with nitrogen gas for 30 min at room temperature, after which the flask was immersed in an oil bath at 70°C with magnetic stirring at 600 rpm. The reaction was led for 12 h. Polymer precipitation was achieved by placing the flask in an ice bath and slowly dripping 40 ml of cold acetone with constant magnetic stirring; the recovered product was filtered and dried at 80°C for 24 h. The CTA agent and initiator calculations were adjusted to a theoretical molecular weight of 70,000 g/mol. A translucent pinkish colored rigid wafer was obtained, 67% of the polymer was recovered, and the conversion was ∼100%. The polymer obtained was characterized by APC, ^1^H NMR, and DLS (Supplementary Figure S12).

### Synthesis of PSS Macro-CTA

For the synthesis of MACRO-CTA of PSS, 9.7 mmol of sodium *p*-styrene sulfonate, 0.08 mmol of S-(thiobenzoyl) thioglycolic acid (TBTGA) as CTA agent, 0.016 mmol of 4,4′-Azobis (4-cyanovaleric acid) as initiator, and 13 ml of a 1:12 aqueous methanol: water solution was added. The synthesis procedure was the same as described in the previous section. The CTA agent and initiator calculations were adjusted to a theoretical molecular weight of 25,000 g/mol. The obtained polymer was characterized by APC and ^1^H NMR (Supplementary Figure S13).

### Synthesis of PSS-b-PS

For the polymerization of poly (styrene-*b*-styrene sulfonate) (PSS-*b*-PS), 0.01 mmol of Macro-CTA PSS, 3.8 mmol of styrene (previously distilled), 0.0071 mmol of 4,4′-Azobis (4-cyanovaleric acid) as initiator, and 12 ml of a 1:2 water: methanol solution was added. The pH of the solution was adjusted to 1 by dropping 1N H_2_SO_4_. The solution was transferred to a sealed magnetic stirrer round-bottom flask with a rubber septum and purged with nitrogen gas for 30 min at room temperature. After, the flask was immersed in an oil bath at 70°C with stirring at 600 rpm and the reaction proceeded for 12 h. The dispersion obtained was a cloudy pinkish-white color that differed from the original transparent pinkish solution. The CTA agent and initiator calculations were adjusted to a theoretical chain extension of 64,500 g/mol or 620 styrenic units. The polymer obtained was characterized by ^1^H NMR and DLS (Supplementary Figure S14).

### Biomimetic Synthesis of Homopolymers and Copolymers doped With PSS, PSS-b-PS, and Co-doping TSA/PSS or TSA/PSS-b-PS

10 mg of Catalyst (Ferrocene or Hematin) and 20 ml of PSS or PSS-*b*-PS solution were added, or using the co-doping ratios assigned in Supplementary Table S3, S4. The rest of the protocol was according to the method described above.

### Raman Spectroscopy

The Raman spectra were acquired on a Horiba Scientific Xplora microRaman; the measurements were carried out using a 532 nm nanoLED at 25 mW (attenuated 10%).

### Real Time Monitoring of UV-vis Polymerization

The UV spectra were acquired in a Cintra GBC spectrophotometer, following the change in absorbance, using deionized water as solvent and quartz cells. In the case of real-time monitoring, the synthesis protocol described above was followed, by measuring periodically the reaction spectra between 300 and 1100 nm.

### Z Potential Measurements

The Z potential was determined with a Microtrac ZETA-Check Zeta Potential Analyzer. The colloidal concentration was 10 mg/ml in deionized water (pH 7 at 25°C).

### Dynamic Light Scattering

The characterization of the size of the synthesized materials was carried out by DLS with a Zetasizer Nano S90 equipment, operating a He-Ne laser at 633 nm and 4mW, and the detection angle was 90°. The diameters of the particles by average intensity and the polydispersity index (PDI) were calculated by means of cumulative analysis according to the ISO13321 standard. The size distribution by intensity was obtained from the function correlation analysis using the general-purpose algorithm included in the instrument software; said algorithm is based on non-negative least squares fit.

### Powder X-Ray Diffraction

The PXRD patterns were acquired on a Brucker D8 Advance Diffractometer with a Cu Kα radiation source (λ = 1.5418 Å). The powdered samples were placed in a standard sample holder. The measurements were made with an interval of 0.02° at a scanning speed of 10°/min from 2θ = 2–82°.

### Infrared Spectroscopy (Fourier Transform Infrared)

Fourier transform infrared spectra were acquired using a Thermo Fischer Scientific FTIR Spectrophotometer in attenuated total reflectance (ATR) mode using a diamond crystal. The sample did not require preparation. The powder is placed on the surface of the glass and the measurement is carried out. The spectra were acquired taking an average of 32 scans with a resolution of 4 cm^−1^ in a range between 400 cm^−1^ to 4000 cm^−1^.

### Measurement of Volumetric Electrical Conductivity

To measure the volumetric resistivity of the materials, 12 mm diameter tablets were prepared on which an electrode was painted with silver varnish in a regular way on both sides. Then, the electrometer tips were placed, and the resistance of the materials was measured. Resistivity was calculated according to the equation VRV = Ω*A/L, where VRV is the volume resistivity in (Ω*cm), Ω is the resistance in Ohms, A is the area of the electrode, and L is the distance between electrodes. The inverse of VR is equal to the volumetric conductivity expressed in S/cm.

## Results

### Polymerization Followed by UV-vis

**FIGURE 1:**
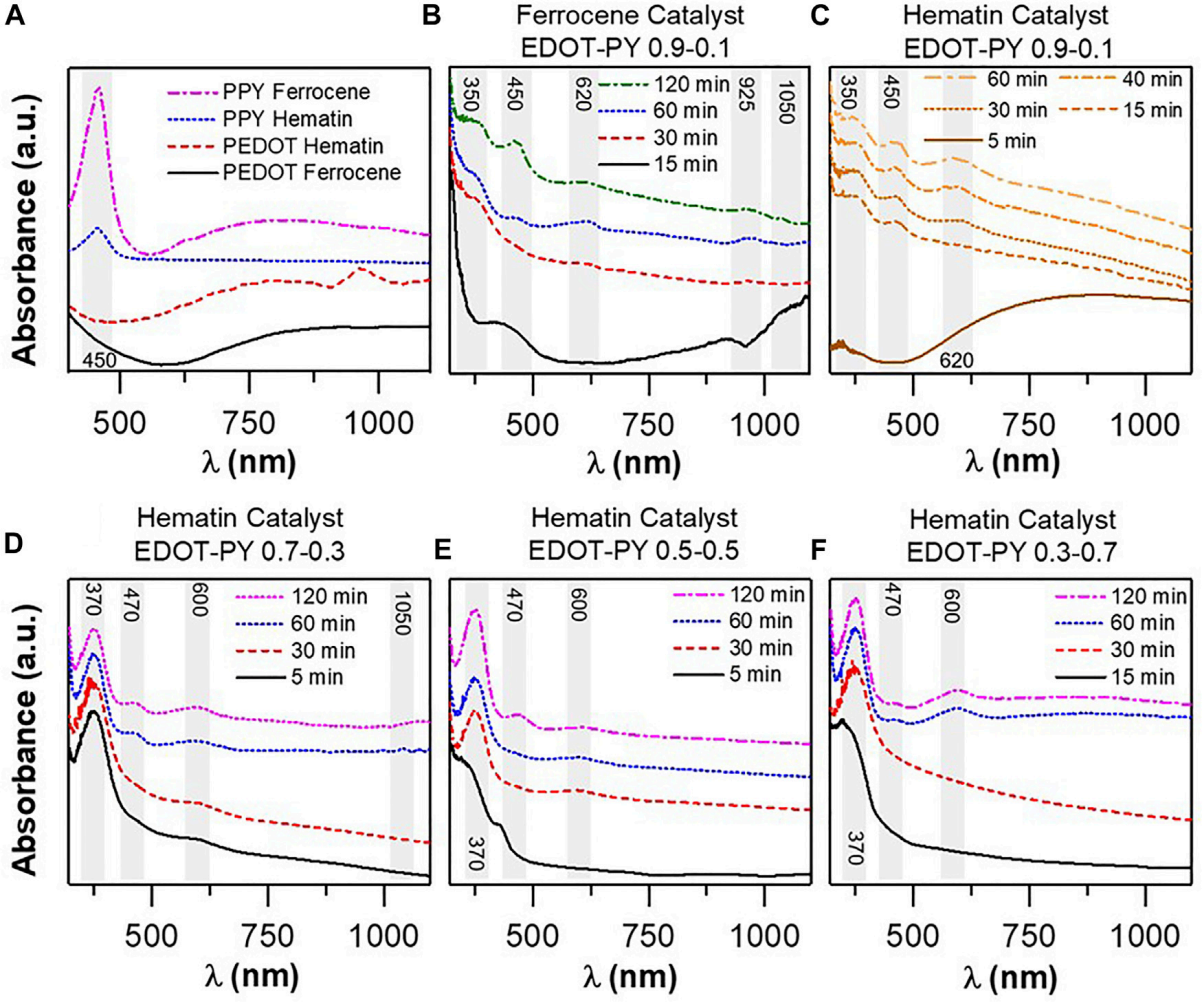
**(A)** UV-vis spectra of biomimetically synthesized PEDOT and PPY homopolymers in the presence of different catalysts, **(B)** UV-vis monitoring of the polymerization of the EDOT-PY 0.9–0.1 copolymer (X molar) catalyzed in the presence of Ferrocene, **(C)** UV-vis monitoring of the polymerization of the EDOT-PY 0.9–0.1 copolymer (X molar) catalyzed in the presence of Hematin, **(D)** UV-vis monitoring of the polymerization of the EDOT-PY copolymer 0.7–0.3 Hematin, **(E)** UV-vis monitoring of EDOT-PY copolymer polymerization 0.5–0.5 Hematin, **(F)** UV-vis monitoring of EDOT-PY copolymer polymerization 0.3–0.7 Hematin.

The analysis of the different synthesized polymeric structures was followed by UV-vis spectrometry. In Figure 1A **,** the UV-vis spectra of PEDOT and PPY homopolymers are presented using ferrocene and hematin as catalysts. In the case of PEDOT synthesized in the presence of ferrocene, the π-π* transition of the thiophene ring is not observed (400–600 nm) (Harri J. Ahonen et al., 2000). This absence could be due to the increasec length and doping of the polymer chain, which causes a bathochromic shift towards energies below 650 nm (>2.1 eV) and leads to an overlap with the low energy polaronic and bipolaronic transitions (800–1300 nm, 1.5–1.1 eV) (Garreau et al., 2001). The sample displays a UV-vis signal distribution typical from a highly doped polymer (Chung et al., 1984). In PEDOT synthesized in the presence of hematin, the bathochromic displacement of the π-π * transition was towards lower energies (∼600 nm), suggesting a change in chain lengths. Regarding the transition whose maximum is centered at 980 nm, it can be considered a polaronic type (Chen and Inganäs, 1996). Both catalysts give rise to highly doped polymers, but the shape of the spectrum for PEDOT catalyzed by hematin suggests a polymer with higher charge mobility. Although it is difficult to conclude as it is not possible to observe the absorptions that extend to the near infrared radiation (NIR) (∼2000 nm), the bipolaronic transitions corroborate the metallic character of the structure (Ahonen et al., 2000).

From the PPY spectra in Figure 1A, an electronic transition is observed at ∼450 nm, which can be associated with the change in the conjugation of the chain and the increase in its length. Typically, the neutral oligomers of PPY present electronic transitions HOMO-LUMO (π-π*) between 300 and 350 nm, however, as the number of pyrrole units increases the absorption maximum shifts towards the red (Okur and Salzner, 2009). It should be noted that in the PPY synthesized in the presence of ferrocene a shoulder at 426 nm is observed, which indicates the existence of chains with a lower number of rings. Bipolaronic or polaronic electronic transitions are also present at higher frequencies (Rahaman et al., 2018) (624 and 1000 nm); such transitions are associated with energy sublevels caused by doping of the structure that decrease the distance between the HOMO and LUMO orbitals. If the degree of doping increases, transitions are observed along the higher wavelengths according to Okur et *al.*, so it is more likely that the charge carriers are associated to polarons, also called diradicals (Okur and Salzner, 2009).

Figures 1B–F show the UV-vis spectra acquired at different reaction times during the polymerization of the EDOT-PY copolymers using four molar ratios: 0.9–0.1, 0.7–0.3, 0.5–0.5, and 0.3–0.7. Figure 1B shows the spectra of EDOT-PY 0.9–0.1 (molar fraction with respect to each monomer); the same reaction conditions described in the methodology were used to monitor polymerization. An aliquot was taken every 15 min and its spectrum was obtained. In the case of the copolymer synthesized in the presence of ferrocene, after 15 min of reaction, four distinguishable electronic transitions appeared with absorption maxima at 350, 450, 925, and 1050 nm. The first one can be attributed to a sub-level of the HOMO-molecular orbital type anti-bond with cationic character that comes from the oxidation of the pyrrole monomer and the formation of oligocations with few rings (Okur and Salzner, 2009). The transition at 450 nm is probably due to the formation of very low molecular weight cationic PEDOT oligomers (Rubio et al., 2001). Regarding the transitions at 925 and 1050 nm, they are sublevels associated with charge carriers of the bipolaric or biradicalic type (Harri J. Ahonen et al., 2000), which indicates that for a short time the monomers have oxidized and started to form chains with different molecular weights. After 30 min, high-energy transitions have undergone displacements towards 400 and 470 nm. The appearance of a new transition with a maximum at 620 nm is observed with an absorption tail that extends towards the NIR. In this region, a bathochromic displacement of the transition from 924 to 965 nm is observed. The 620 nm transition is interesting since it seems to indicate the appearance of new polaron species of EDOT-PY within the polymeric backbone according to Astratine et al. (2014) Our results offer strong evidence of the formation of an EDOT-PY copolymer, since there are three transitions observed experimentally by Astratine et *al.* however, the positions of the absorptions of our copolymer are shifted towards the red with respect to the findings of the previous studies. On the other hand, a copolymer with very similar characteristics to the one prepared here was synthesized by Tarkuc et *al*. from a thiophene-pyrrole-thiophene trimer and EDOT Tarkuc et al. (2008). Such a copolymer exhibits the three transitions also observed in our copolymer, although with differences in the transition peaks position (320, 470, and 800 nm). It can be considered that an EDOT-PY copolymer was obtained with a distribution not completely alternated but formed by blocks of multiple EDOT rings and short polypyrrole segments (proposed structure Supplementary Scheme S1 Support Information). The results described when using the catalyst ferrocene are highly concomitant with the hematin catalyst as can be seen in Figure 1C, which spectra presents the same transitions as stated above. The UV-vis spectra acquired at different reaction times during the polymerization of the 0.7–0.3 EDOT-PY copolymer (mole fraction with respect to each monomer) is shown in Figure 1D. After 15 min of reaction, three electronic transitions with maximums of absorption at 370, 470, and 600 nm were detected. The first is the most prominent of all. The increase in intensity when changing the molar ratio of EDOT-PY is consistent with that reported by Astratine et al. (2014). The transition at 470 nm has low intensity, which indicates a lower contribution of such segment to the electronic configuration while the polaron transition at 600 nm also exhibits low intensity. At 30 min of reaction the trend of the intensity observed in the 370 nm band remains almost constant. After 60 min the intensity of the transitions at 470 and 600 nm increases, which means an increase in the molecular segments exhibiting the different energy sublevels (Rubio et al., 2001; Okur and Salzner, 2009). A transition close to 1050 nm appears and is ascribed to the overlapping of biradicals. In Figures 1E,F (EDOT-PY 0.5–0.5 and 0.3–0.7) the same absorptions are observed. The 370 nm electronic transition associated with the polaron of the pyrrole segment maintains a higher intensity relationship with respect to the other two transitions, which is an obvious consequence of the increase in the percentage of pyrrolic rings in the structure. These findings are consistent with the mentioned literature. Detailed discussion of the UV-vis characterization of the EDOT-PY copolymer doped with PSS and PSS-PS can be found in Supplementary Figure S2 of the support information. Spectra like those of doping with TSA but with differences associated with the increased dispersibility and interaction with the polyanionic template. In general, with the appearance of the mentioned bands, we can confirm the successful synthesis of the studied doped copolymers. The UV-vis study gives concrete and solid evidence of the formation of the EDOT-PY copolymer, whose stable structure does not depend on the type of catalyst but is dependent on the molar ratio of the monomers used.

**FIGURE 2:**
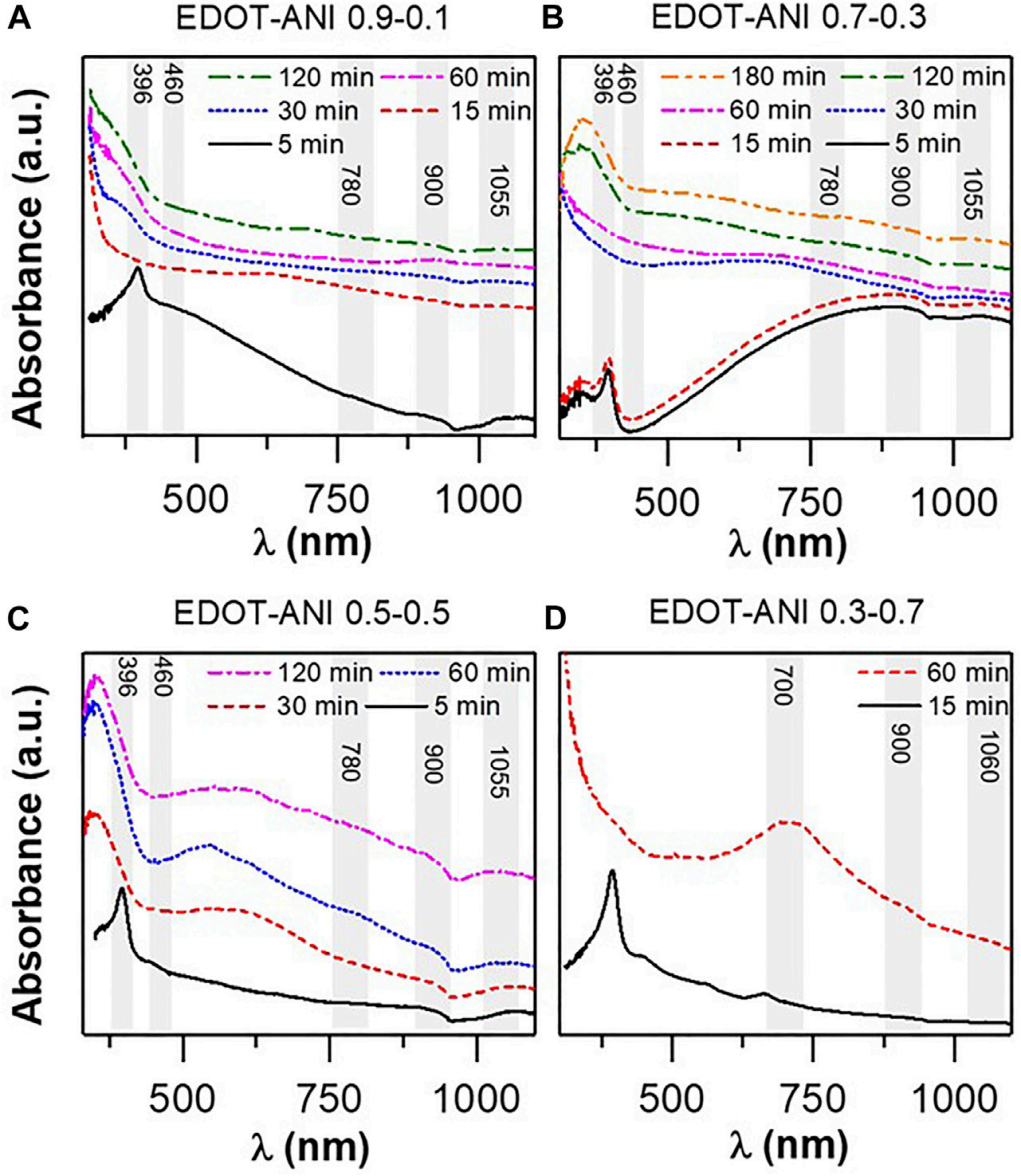
UV-vis spectra: **(A)** EDOT-ANI 09–0.1 polymerization monitoring, **(B)** EDOT-ANI 0.7–0.3, **(C)** EDOT-ANI 0.5–0.5, **(D)** EDOT-ANI 0.3–0.7.

Supplementary Figure S1 shows the UV-vis spectrum of the base PANI that was acquired in NMP. A band around 319–329 nm is observed that corresponds to the π-π* transition of the aromatic aniline ring. The broad band at 616–628 nm agrees with the n-π* transition of quinine-imine groups, which is a benzenoid to quinoid exitonic transition (Pruneanu et al., 1999). Figure 2A–C contain the spectra obtained during the monitoring of the polymerization reaction of the EDOT-ANI copolymer catalyzed by hematin (Hematin was the only catalyst evaluated because ferrocene did not efficiently catalyze the polymerization reaction of PANI). During the first 5 min of the reaction, the appearance of electronic transitions was observed. The first transition occured at high frequency with an absorption maximum at 396 nm, which can be assigned to the formation of polarons of the adjacent PANI molecular segments (Xia et al., 1994). A shoulder at ∼460 nm was observed for this transition, which can be ascribed to the polaron-type electronic transition that occurs in the molecular segments of EDOT rings (Rubio et al., 2001). The red shift of these transitions is due to the change in conjugation and state of oxidation of the growing chains. The transitions expected for the neutral segments are not observable. There are also faint center transitions at 780, 900, and 1055 nm, associated with the formation of bipolarons in the copolymer chain (Garreau et al., 2001). While for the EDOT-ANI 0.3–0.7 copolymer (Figure 2D), only three transitions are observed at 700, 900, and 1060 nm. Similar characteristics can be observed in EDOT-ANI copolymers doped with PSS and PSS-PS (Supplementary Figure S3).

### Raman Spectroscopy

**FIGURE 3:**
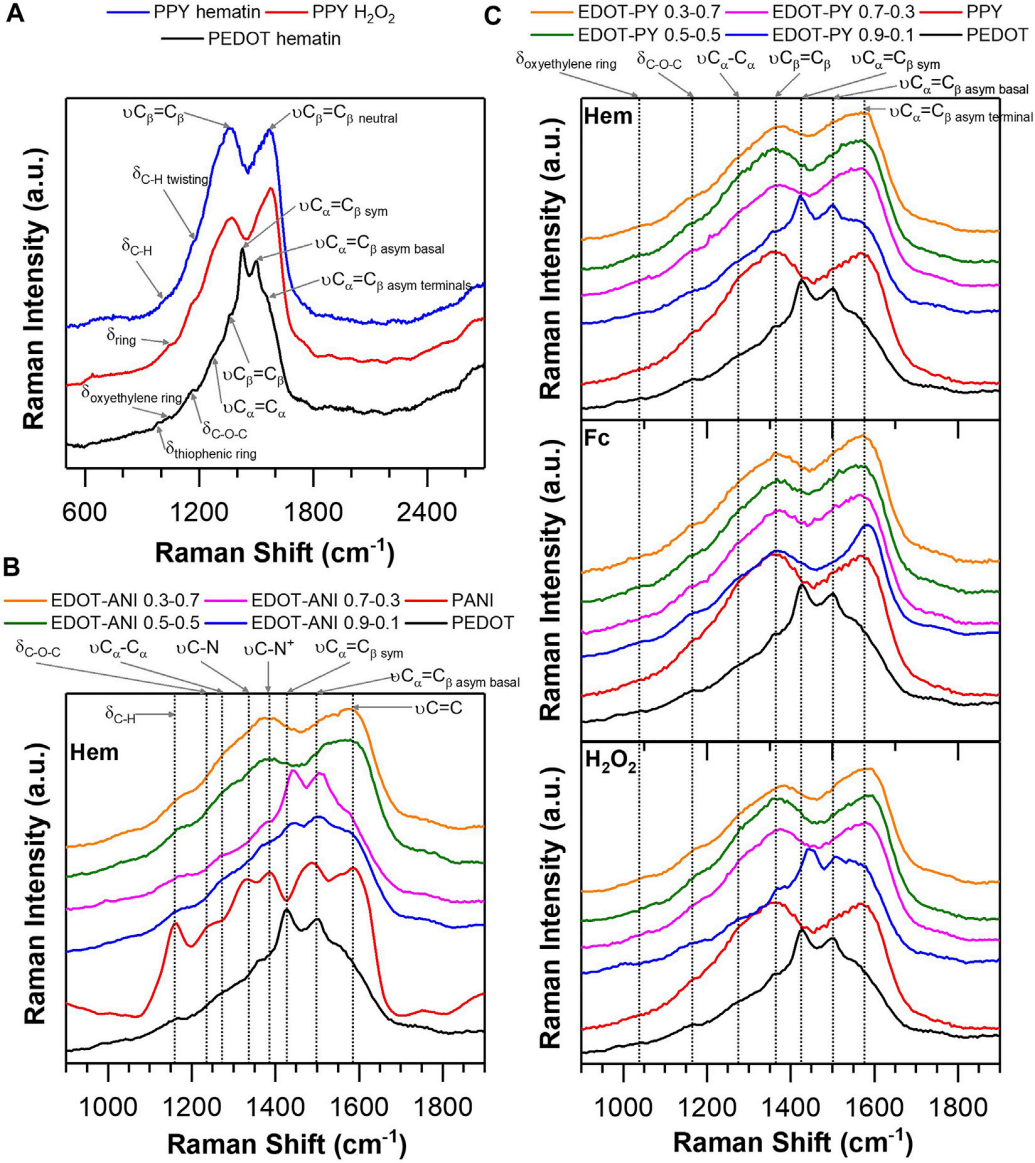
**(A)** Raman spectra of PEDOT and PPY obtained using different biomimetic catalysts, **(B)** Raman spectra of PANI, PEDOT, and EDOT-ANI copolymers with different molar ratio obtained by biomimetic synthesis using hematin as catalyst, **(C)** Raman spectra of copolymers obtained using different biomimetic catalysts: EDOT-PY/Ferrocene, EDOT-PY/Hematin, EDOT-PY without catalyst.

The Raman spectra obtained using a laser of λ = 532 nm is shown in Figure 3A. For the PPY obtained in the presence of the biomimetic catalyst hematin, four main signals are distinguished. At high frequencies the stretching of the benzenoid form of the pyrrole ring is observed [1570 cm^−1^, νCα = Cβ, signal also associated with the cationic form and not the neutral (Kepas et al., 1978)]. The signal at 1370 cm^−1^ is associated with the stretching of the beta carbons of the pyrrole ring (νCβ = Cβ). The signal at 1170 cm^−1^ is an out-of-plane deformation, and it is overlapped with more energetic virtual states, which comes most likely from the twisting of the CH bond in the beta carbon of the ring. Another small shoulder is located at 1020 cm^−1^, which may correspond to the deformation in the plane of the C-H bond. In general, all the signals observed correspond to those reported for polypyrrole in the literature (Arteaga et al., 2013; Varade et al., 2013; Gniadek et al., 2014; Herrera Herrera et al., 2018). In the case of polypyrrole obtained in the absence of catalyst, the Raman spectrum reveals that hydrogen peroxide gives rise to polypyrrole with spectral characteristics very similar to that obtained by enzymomimetic catalysis. It is remarkable that the stretching of β carbons decreases in intensity for PPY-hematin. This observation could indicate a decrease in the quinoid form within the chain that results in a loss of conjugation and electronic delocalization in the molecular skeleton (Kępas et al., 2007). However, the observation is not conclusive since in both structures the asymmetric stretching νCα = Cβ is moved into a lower frequency than what is typically reported for PPY driver (approx. 30 cm^−1^). This indicates a lower degree of conjugation as the neutral segments predominate associated. Vibrational transition could mean that both polymers have low electrical conductivity (Santos et al., 2007). Regarding PEDOT (Figure 3A), its Raman spectrum shows at least eight distinguishable signals (they can probably be defined with precision after deconvoluting). At higher frequencies the asymmetric alpha-beta stretching of the terminal rings of the chain is observed at 1556 cm^−1^ (νCα = Cβ) (Xu et al., 2017). At 1503 cm ^−1^ an intense and narrow peak is observed that corresponds to the alpha-beta asymmetric stretching but of intermediate rings of the chain (Dunst et al., 2018). The peak of 1427 cm^−1^ corresponds to the symmetric alpha-beta stretching of the ring carbons (νCα = Cβ) (Funda et al., 2016). The shoulders at 1356 and 1260 cm^−1^ are associated with stretching of beta-beta and alpha-alpha carbons, respectively (Kim et al., 2014). Finally, the low frequency signals at 1150, 1040, and 992 cm^−1^ are ascribed to deformations of the type: COC bond, deformation in the plane of the oxyethylene ring, and deformation in the plane of the thiophene ring (Yoo et al., 2015; Funda et al., 2016; Dunst et al., 2018). Figure 3C shows the Raman spectra acquired for EDOT-PY copolymers synthesized biomimetically using ferrocene and hematin as catalysts. A blank without catalyst was also characterized to analyze if the effect in the oxidative synthesis was promoted by hydrogen peroxide. Although there are various works in the area, there is no such comparison so far. In the case of the ferrocene-catalyzed EDOT-PY copolymer, the evolution of the spectra is observed by varying the molar ratio of the monomers. In the four copolymers the existence of six vibrational energy changes associated with the movements described in the previous section is observed. When studying the 0.9–0.1 M ratio in detail, there is a decrease in intensity and overlap of the signals of the PEDOT segments in the copolymer associated with symmetric and asymmetric alpha-beta stretching of the thiophene ring (Funda et al., 2016). This suggests a change in the oxidation state of the chains (Santos et al., 2007), which coincides with our observations from UV-vis spectroscopic analysis, that indicate a small introduction of pyrrolic rings to the PEDOT chain decreases the conjugation of long segments that can be interpreted at the molecular level as the appearance of intermediate pyrrole oligo segments to longer segments of PEDOT affecting their electronic distribution, planarity of the structure, and doping (Okur and Salzner, 2009; Wang X. et al., 2015). The 0.9–0.1 copolymer catalyzed with hematin exhibits two peaks at 1420 and 1503 cm^−1^, which are associated with symmetric and asymmetric alpha-beta stretching of the thiophene ring (Funda et al., 2016). Regarding the target without catalyst in the ratio 0.9–0.1, it unexpectedly shows a spectral pattern very similar to that observed in catalysis with hematin, but the widening of the signals between 1500 and 1550 cm^−1^ indicates a loss of conjugation and the predominance of segments of neutral polypyrrole (Santos et al., 2007). In general, the 0.9–0.1 copolymer catalyzed by hematin appears to show the spectral characteristics closest to PEDOT. From copolymer 0.7–0.3 to 0.3–0.7, the spectral properties of the three structures are very similar to PPY, with small shoulders associated to the thiophene segments. The most remarkable result from Raman analysis is that the spectral patterns of the different copolymers vary very slightly depending on the type of catalyst used from the 0.7–0.3 M ratio. This is consistent with our findings in UV-vis spectroscopy studies. However, based on spectroscopic evidence, it is speculated that at higher molar ratios of pyrrole in the copolymer, the typical PPY chain defects will appear (crosslinking, breaking, branching), which affect the electrical conductivity.

Regarding the Raman spectrum of PANI (Figure 3B), the following vibrational transitions of the polymer can be identified: 1585 cm^−1^ νC = C of the benzenoid ring, 1550 cm^−1^ νC = C associated with emeraldine segments, 1486 cm^−1^ νC = N of the ring quinoid, between 1392 and 1332 cm^−1^ νC-N^+^ polarons located in chains of different lengths, 1243 cm^−1^ νC-N of the amine, 1158 cm^−1^ deformations outside the plane of CH, and 811 cm^−1^ deformations in the plane of the ring (Rohom et al., 2014; Wang L. et al., 2015; Kulandaivalu et al., 2015, 2016). When analyzing the EDOT-ANI 0.9–0.1 copolymer (Figure 3B), the following bands are identified: 1580 cm^−1^ νC = C associated to the benzenoid ring of the polyaniline molecular segments, 1500 cm^−1^ and 1420 cm^−1^ are vibration modes assigned to asymmetric stretching and symmetric of the alpha-beta carbons of the PEDOT (Dunst et al., 2018) segments, as well as bands at 1360, 1270, 1170, and 1048 cm^−1^ assigned to beta-beta carbon stretching and alpha-alpha stretching of thiophene rings, deformations in the CH plane of both the thiophene and benzenoid rings of aniline, and deformations in the plane of the oxyethylene ring of EDOT (Yoo et al., 2015; Funda et al., 2016; Dunst et al., 2018). The copolymer 0.7–0.3 exhibits the same bands with similar intensities, and the PEDOT-type spectral pattern is preserved, which suggests a greater delocalization of charges on the chains and a greater structural order of the copolymer (Wang X. et al., 2015). In copolymers 0.5–0.5 and 0.3–0.7, two broad bands are observed between 1600 and 1500 cm^−1^ and between 1350 and 1450 cm^−1^, which are the product of the overlap of vibrational modes with similar intensities. Both copolymers exhibit contributions of segments of PANI and PEDOT, but no band is perfectly resolved. This seems to indicate that the degree of conjugation decreases due to the formation of alternating short molecular segments of aniline and EDOT. Raman study offers solid information on the confirmation of the copolymer EDOT-ANI.

### FTIR Spectrometry

**FIGURE 4:**
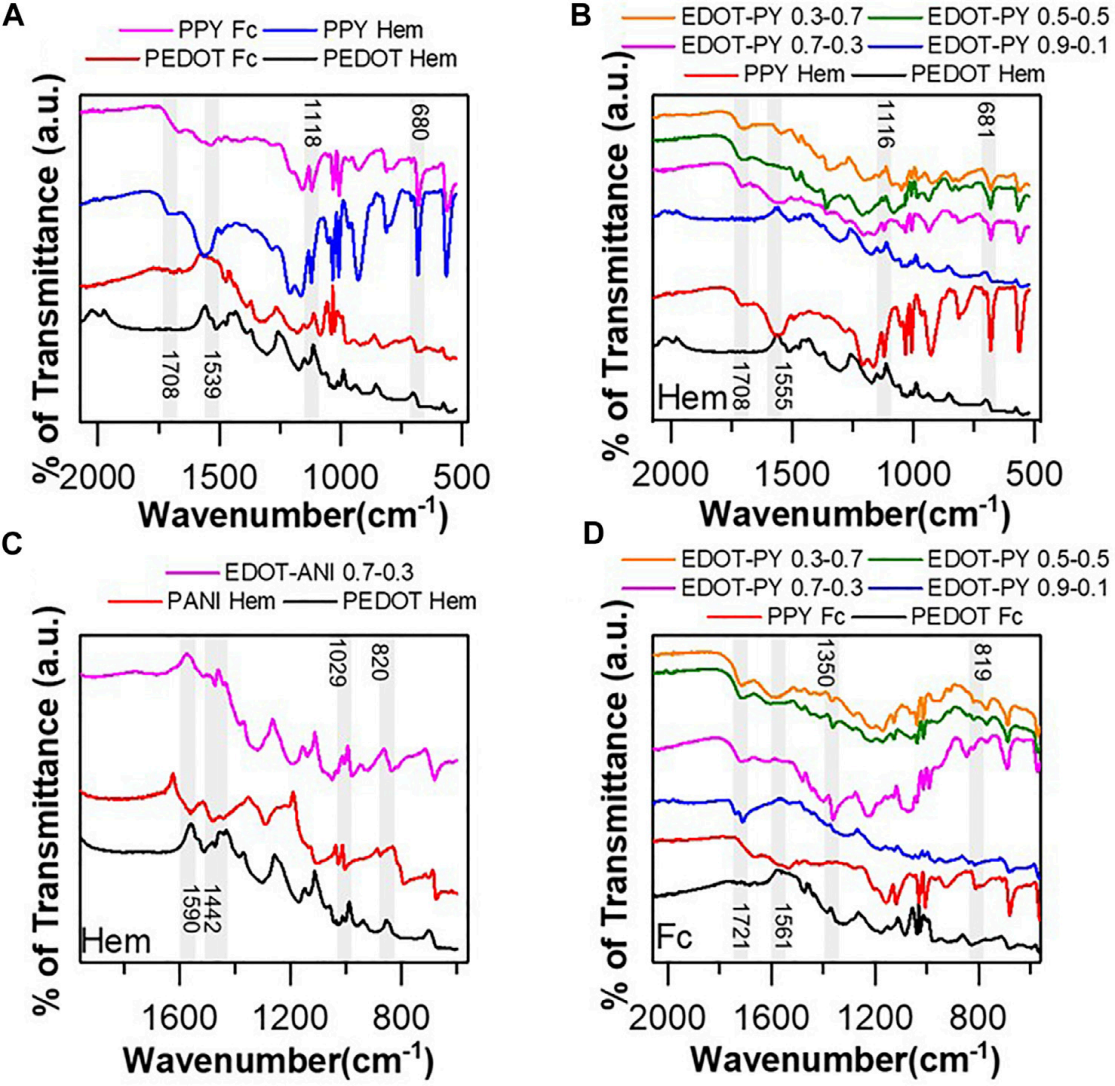
**(A)** FTIR spectra of PEDOT and PPY synthesized biomimetically using ferrocene and hematin as catalysts, **(B)** FTIR spectra EDOT-PY copolymers Hematin-catalyzed Biomimetic synthesis, **(C)** FTIR spectra copolymers EDOT-ANI biomimetic synthesis catalyzed by Hematin, **(D)** FTIR spectra EDOT-PY copolymers Ferrocene-catalyzed biomimetic synthesis.

The FTIR spectra of the samples are shown in Figure 4 and Supplementary Table S5 (support information) and collects the main bands identified in the different polymers analyzed. In PEDOT-Hematin (Figure 4A), the appearance of a band related to asymmetric stretching C=C of the ring (Sandoval et al., 2015) is observed at 1539 cm^−1^, which does not appear in PEDOT Fc. This observation could mean a decrease in conjugation in the chain linked to defects such as branching and crosslinking, which will affect the conductivity of the polymer. Figures 4B,D show the FTIR spectra of the EDOT-PY copolymers: 0.9–0.1, 0.7–0.3, 0.5–0.5, and 0.3–0.7 biomimetically synthesized in the presence of Hematin and Ferrocene. Supplementary Table S5 condenses the bands identified for each copolymer. Copolymer 0.9–0.1 (Figures 4B,D) shows some of the same bands obtained for its homopolymers, corroborating the contributions of both the chemical structures and the polymeric skeleton, in agreement with that reported by Bruno et al. (2006). The molar ratios 0.7–0.3, 0.5–0.5, and 0.3–0.7 show bands of both PEDOT and PPY confirming the existence of copolymers. The same can be observed when the copolymers are doped with PSS and PSS-PS (for more details review FTIR section support information). FTIR spectroscopy allows us to conclude that neither the catalyst nor the type of dopant exert a significant change in the chemical structure of the copolymer. Figure 4C shows the FTIR spectra of PEDOT, PANI, and EDOT-ANI copolymers (molar ratio 0.9–0.1 and 0.7–0.3). Supplementary Table S6, S7 shows the assignments of the bands identified in the spectra. As analyzed before, the PEDOT spectrum presents the bands previously reported in the literature. In the case of PANI, the main bands coincide with the conventional vibrational modes reported for this polymer (Trchová and Stejskal, 2011). In the case of EDOT-ANI copolymers, the spectra show bands related to both rings, confirming the existence of the copolymer. Likewise, the products with a molar ratio between 0.9–0.1 and 0.7–0.3 show identical spectral patterns. Copolymers doped with PSS were found to show bands and intensity very similar to that observed with doping with TSA. As discussed here, this observation is evidence of chemical structures with specific vibrations, which is consistent with our observations from UV and Raman spectrometry characterization, giving a broader picture of the chemical and structural nature of the synthesized copolymers.

### X-Ray Diffraction

**FIGURE 5:**
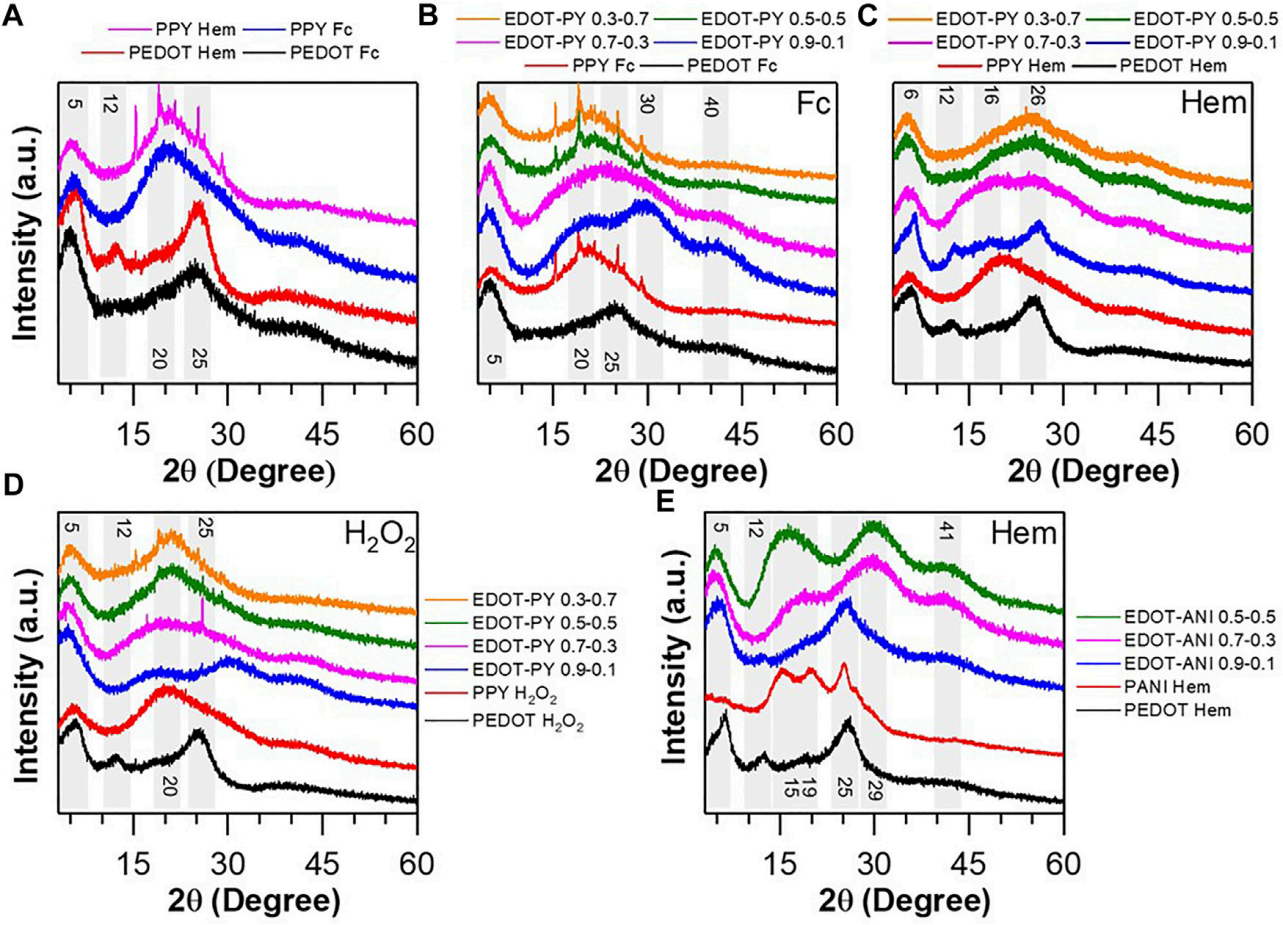
Diffractograms of homopolymers and copolymers: **(A)** PEDOT and PPY using Hematin and Ferrocene catalysts, **(B)** EDOT-PY copolymers with Ferrocene catalyst, **(C)** EDOT-PY copolymers using Hematin catalyst, **(D)** EDOT-PY copolymers without catalyst, **(E)** Diffractogram of PEDOT, PANI and EDOT-ANI copolymers with different molar ratios doped with TSA and using Hematin as catalyst.

The results of the X-ray diffraction analysis of the synthesized polymers are shown in Figure 5. The PEDOT obtained using ferrocene as a catalyst (Figure 5B) shows a wide diffraction at 2θ = 25° associated with the d (010) (Wang X. et al., 2018) plane that describes the stacking distance between adjacent chains due to π-π interactions. Another diffraction is observed at 2θ = 5° associated with the d (100) plane that is related to the interchain distance on the XY (Zhou et al., 2015) plane, and which tends to increase when doping the structure. It has been previously reported that, as the ion is larger and the amount of doping increases, the plane tends to increase its distance (Wang X. et al., 2018). Regarding the PEDOT obtained using hematin, the following diffractions are observed: 2θ = 5.8, 12.3, 15.1, 18.3, and 25.1°. These can be assigned to plane d (100) of the distance between interchain segments on the XY plane with associated ions, d-plane (100) of the distance between segments interchain, plane d (200) associated with interchain distributions with greater packing, the diffraction at 2θ = 18.3° is associated with the stacking of the TSA, and finally the plane d (010) assigned to the stacking of chains, respectively (Zhou et al., 2015; Bahry et al., 2018; Wang X. et al., 2018; Kim et al., 2019). The peaks are narrower than with the ferrocene catalyst. It is deduced that the Hematin enzymomimetic catalyst favors the formation of a structure with greater molecular order in the long range, which could mean improvements in electrical conduction since highly amorphous tertiary structures do not favor the intermolecular load hopping (electronic hopping). When analyzing the PPY diffractograms, two main diffractions centered at 2θ = 5.3° and 20.5° are observed (Figures 5B,C), which can be associated with plane d (100) and plane d (002) (Sanches et al., 2015). The first describes the interchain distance on a two-dimensional plane, while the second refers to the stacking of chains. In both cases, they are wide, which means a high degree of disorder of the polymer (Kim et al., 2019). In the case of PPY-ferrocene, there are four parasitic signals observed protruding from the amorphous peak. The most reasonable assumption is to consider them as diffractions belonging to the TSA. Figure 5B shows the diffractograms of the EDOT-PY copolymer synthesized using Ferrocene as a catalyst. For the 0.9–0.1 M ratio, four very wide-centered diffractions are seen at 2θ = 4.9°, 20.5°, 29.9°, and 40.5°. The diffraction at 2θ = 29.9° is assigned to the stacking of chains of the copolymer (Wang X. et al., 2018); however, its width indicates an increase in the degree of amorphousness of the phase, which agrees with some theoretical predictions that consider loss of planarity in the copolymers that it entails an increase in the degree of disorder of the structure (Kamran et al., 2015). The wide diffraction centered at 2θ = 40.5° is difficult to assign but is probably related to very compact inter-chain or inter-chain and dopant arrangements (Marutaphan et al., 2015). The copolymer 0.7–0.3 exhibits three very wide principal diffractions. The first at 2θ = 4.9° is related to the interchain distance in the XY plane. The second centered at 2θ = 24.5° is extremely wide and implies a high degree of disorder of the structure. Copolymers with ratios 0.5–0.5 and 0.3–0.7 have very similar diffractograms, with two main diffractions: 2θ = 4.9 and 21.4°. When considering the diffractogram of the 0.9–0.1 copolymer but with the Hematin catalyst (Figure 5C), the following diffractions, 2θ = 6.2, 12.6, 16.5, 19, and 26.2°, are observed, which correspond to the planes described previously, but with small increases in angle that suggest a decrease in interplanar distances; likewise the peaks are narrower. These observations suggest an increase in the order of the chains (Wang X. et al., 2018). The copolymer 0.7–0.3 synthesized using hematin has the same characteristics as with the use of Ferrocene. This shows that the molar ratio has an important effect on the crystalline structure of the copolymer, and as the amount of EDOT in the structure decreases, the increase in amorphousness in the products takes place. The copolymers 0.5–0.5 and 0.3–0.7 obtained using hematin as a catalyst have similar characteristics to each other, and they are also like the copolymers 0.5–0.5 and 0.3–0.7 using Ferrocene as a catalyst. They also have similarities to the diffractograms of the EDOT-PY copolymers doped with PSS, which become more amorphous due to the presence of the polyanion (Anothumakkool et al., 2015) (Supplementary Figure S6). The XRD analysis showed that the catalyst has small effects on the crystalline phase, especially when the concentration of EDOT in the copolymer is high. From the ratio 0.7–0.3 the effects are not significant and rather suggest that the structure of the product depends on the molar fraction of pyrrole, giving rise to more amorphous copolymers. The X-ray diffraction results are consistent with the Raman and UV-vis characterization in terms of catalyst not influencing on the structure. X-ray characterization provides strong evidence that the molar ratio has a dominant effect on the structural characteristics of the product.

The diffractograms of PEDOT, PANI, and copolymers of EDOT-ANI doped with TSA are shown in Figure 5E. When analyzing the diffractogram, the peaks that appear at 2θ = 15, 20, and 26°, correspond to the planes (011), (020), and (200) of NIBP; such reflections are characteristic of the emeraldine salt structure (Roy et al., 2002; Zhang et al., 2010; Mostafaei and Zolriasatein, 2012). For the copolymer in molar ratio 0.9–0.1, broad peaks centered at 2θ = 4.9, 12.5, 19, and 25.6° are observed. The first diffraction can be attributed to the interchain distance between adjacent segments of stacked groups and ions; the second is probably the distance interchain of adjacent segments, but without intermediate ions; the third is associated to the stacking between ions; and the last is ascribed to the stacking of chains of the copolymer (Zhou et al., 2015; Bahry et al., 2018; Wang X. et al., 2018; Kim et al., 2019). This diffractogram is closer in positions and interplanar distances to the pure PEDOT. Therefore, the 0.9–0.1 relationship does not seem to significantly affect the crystallinity of the product. On the other hand, in the 0.7–0.3 copolymer four very wide diffractions are observed centered on 2θ = 4.7, 18.5, 29.6, and 41.3°. Different from previous observations, the diffraction assigned to the interchain stacking decreases its interplanar distance, which may be due to the increase in the interaction force between rings (Martinez-Cartagena, 2017). Diffraction at 2θ = 41.3° indicates very compact associations between chains that give rise to configurations with low packing factor (Kwon and McKee, 2000). The copolymer 0.5–0.5 exhibits a diffractogram very similar to the previous ones, such as the EDOT-ANI copolymers doped with PSS and PSS-PS (Supplementary Figure S7). XRD characterization is useful to study the changes in the solid-state arrangement of the copolymer according to the molar fraction of monomers used. The results agree with the previous spectroscopic data, allowing to conclude that the molar ratio used has an important effect on the order in the solid state. Decreasing the molar fraction of EDOT increases the disorder of the structure, obtaining more amorphous copolymers.

### XPS Analysis

**FIGURE 6:**
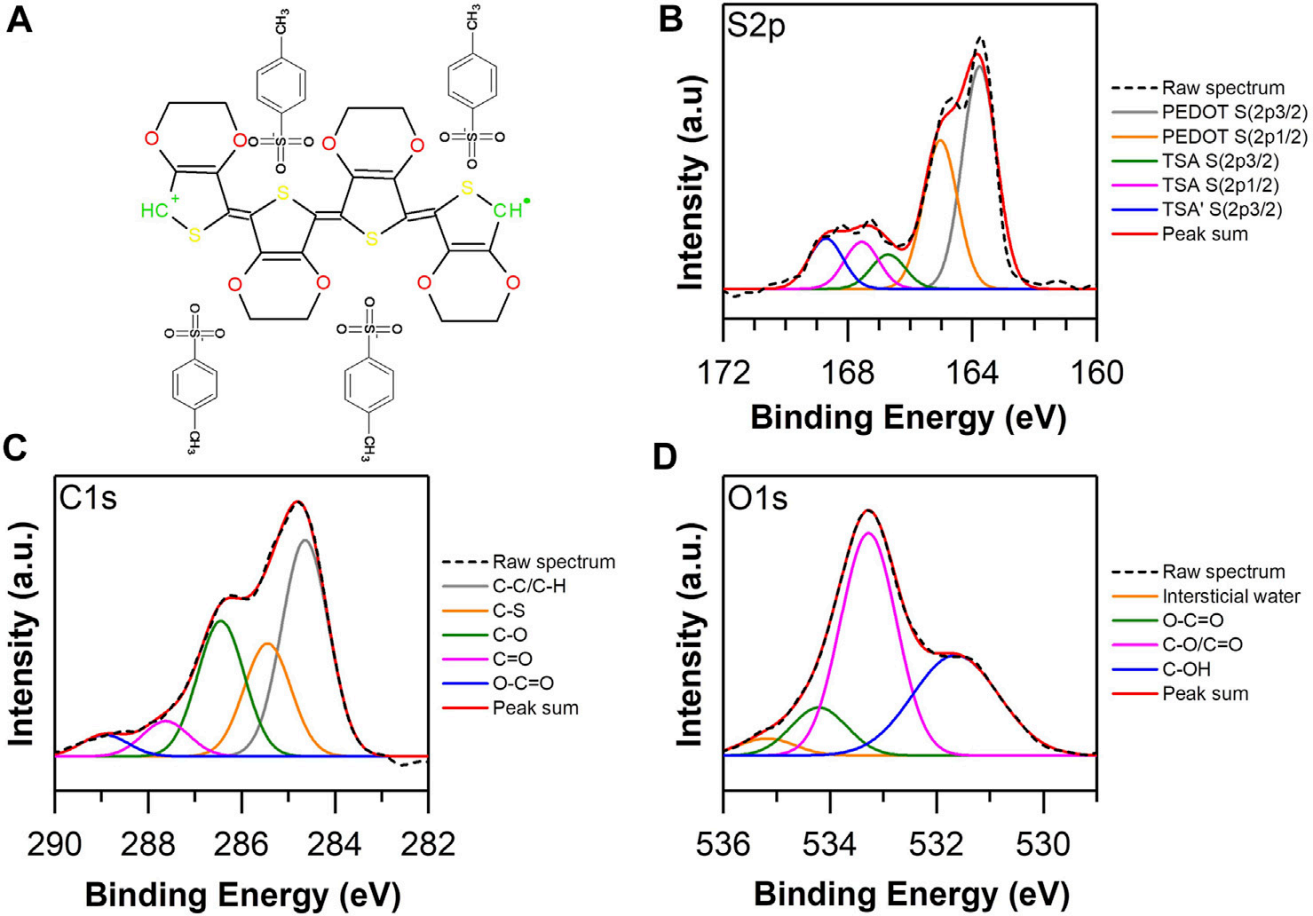
**(A)** Structure of PEDOT: TSA. High resolution XPS spectra of biomimetically synthesized PEDOT TSA: **(B)** S 2p, **(C)** C 1S and **(D)** O 1s.

The X-ray emitted photoelectron spectroscopy (XPS) study was performed to examine the composition and chemical bonds present in the structure of EDOT-PY homopolymers and copolymers as well as EDOT-ANI, to determine the chemical composition of the surface of the bulk materials. The comparison between the chemical structure of homopolymers and copolymers provides a rational basis to establish the structural character of the different synthesized copolymers, which is fundamental to complement other spectroscopic and electrochemical characterizations performed on these materials. Figure 6B shows the high-resolution spectrum S 2p of PEDOT: biomimetically synthesized TSA using hematin as a catalyst, according to the general spectrum (Supplementary Figure S8).

A sulfur concentration of 6.3% was quantified on the surface of the sample analyzed. On the other hand, the high resolution deconvolved spectrum contains five possible bonds, which were assigned to 163.8 and 165.2 eV (Wang et al., 2017), which are associated with S thiophene of the molecular structure of the PEDOT, while the bonds at 166.7, 167.6, and 168.7 eV (Fabretto et al., 2009; Wang M. et al., 2016) are associated with the S atom of the TSA structure that dopes PEDOT chains. An approximate and reliable way to quantify the degree of oxidation or doping of the chains of PEDOT is to study the relationship between the areas of binding energies between PEDOT and TSA. In this case, a 3:1 ratio was obtained; it has been reported in the literature that the ideal doping is 4:1. However, the relationship found is an indicator of a high degree of doping of the synthesized polymer, being a parameter that predicts high conductivity of the polymer in bulk (Wang et al., 2017). Regarding the deconvolved high resolution spectrum of C 1s (Figure 6C), five possible bonds were obtained, assigned as follows (Fabretto et al., 2009; Wei et al., 2015; Deng et al., 2017; Abdul Razzaq et al., 2019): 284.5 eV to the C sp^3^ bond, 285.4 eV to CS, 286.4 is associated with COC, 287.6 eV to carbonyl, and 288.9 a possible ester. The deconvolved high resolution spectrum of O 1s (Figure 6D) contains four probable bonds (Gordovskaya et al., 2014; Lin et al., 2016; Mowbray et al., 2016; Filippov et al., 2018): 531.6 eV associated to C-OH bond, 533.3 eV to CO/C=O, 534.2 eV attached to ester, and 535.2 eV to pore water.

**FIGURE 7:**
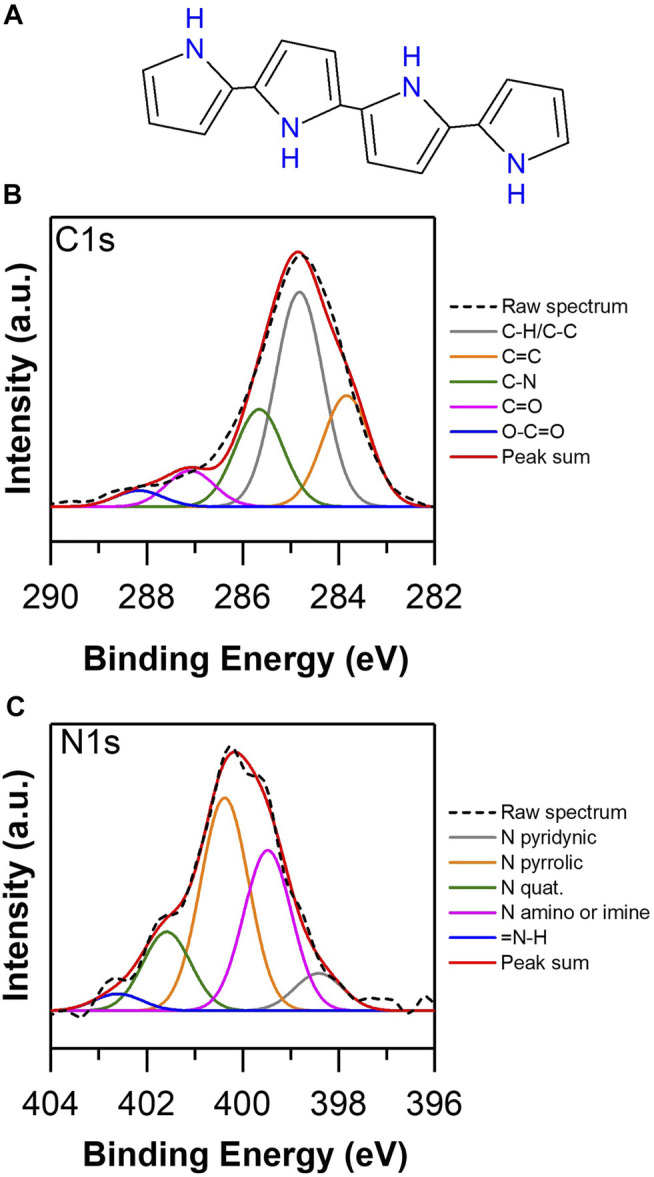
**(A)** Structure of PPY. High resolution XPS spectra of biomimetically synthesized PPY TSA: **(B)** C 1s and **(C)** N 1s.

According to the surface analysis performed on the PEDOT TSA, a high degree of doping of the structure was observed, which indicates a conjugated structure with polarized regions that provide electronic conditions to increase the electrical conductivity of the polymer in bulk. Figure 7 shows the high resolution spectra of C 1s and N 1s of the Polypyrrole TSA; the bonds attached to carbon are 283.7 eV to C sp^2^, 284.85 eV to C sp^3^, 285.65 to CN, 287 eV to carbonyl, and 288.3 eV to carboxylate or ester, while the probable bonds of N are (Gence et al., 2007; Patil et al., 2018; Ghosh et al., 2019; Huang et al., 2019; Šetka et al., 2019) 398.3 eV associated to pyridine N, 399.5 eV to pyridyl-type N, 400.4 eV is ascribed as pyrrolic N, 401.58 eV to quaternary N, and 402.51 eV to = NH. Considering the chemistry observed on the surface of PPY TSA, the correlation between the abundance of C sp^3^ bonds with respect to the C sp^2^ and the abundance of pyrrolic N in the structure show that the degree of conjugation is probably below the ideal amenity of a 17% oxygen concentration in the structure (Supplementary Figure S9), giving clear indications that the biomimetically synthesized PPY presents unsatisfactory electronic characteristics related to the increase of the gap between HOMO/LUMO (Wang et al., 2017), ergo minor conducti electrical life.

**FIGURE 8:**
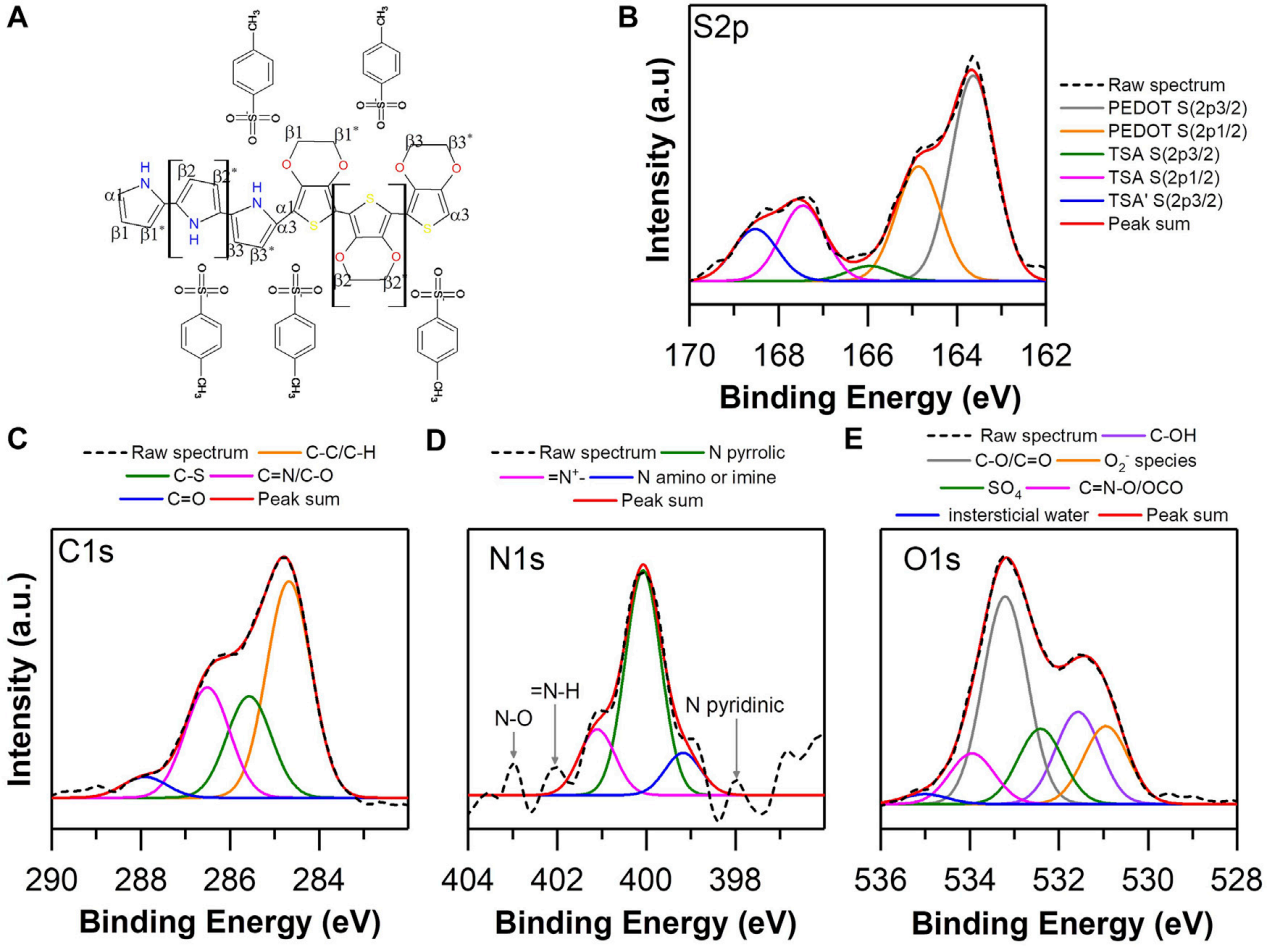
**(A)** Proposed structure of EDOT-PY TSA Copolymer, High Resolution XPS Spectra of EDOT-PY 0.7–0.3 TSA: **(B)** S 2p, **(C)** C 1S, **(D)** N 1s and **(E)** O 1s.

The high-resolution spectra of S, N, C, and O of the copolymer EDOT-PY 0.7–0.3: TSA are presented in Figure 8, the spectrum of C 1s (Figure 8C) exhibits different chemical species assigned as: 284.5 eV to C sp^3^, 285.65 to CS, 286.7 eV to carbonyl/C=N, and 288.2 eV to carboxylate or ester. The high-resolution S 2p spectrum shows five types of links ascribed as 163.8 and 164.9 eV associated with the thiophene S of the molecular structure of EDOT, while the bonds at 166, 167.2, and 168.4 eV are associated with the S atom of the TSA sulfone (Figure 8B). There is a shift of these three binding energies with respect to PEDOT TSA, probably due to a different chemical environment caused by the EDOT-PY structure. Unlike PEDOT TSA, the ratio between areas of binding energies of the S species present does not correspond to the total doping of the structure, since only 70% of the molar composition of the copolymer is based on EDOT. However, it provides useful information to know the approximate level of doping of the EDOT copolymer. PY 0.7–0.3 copolymer ratio was calculated considering only the contribution of EDOT and was equal to 3:1 EDOT: TSA, which coincides with the ratio of PEDOT:TSA. Therefore, it could be inferred that the doping level is relatively high. However, the doping level of the pyrrole units is unknown. The high-resolution spectrum of deconvolved N 1s (Figure 8D) allows the identification of at least six types of nitrogen (Gence et al., 2007; Patil et al., 2018; Ghosh et al., 2019; Huang et al., 2019; Šetka et al., 2019): 398 eV associated with pyridine N, 399.5 eV with N amine, 400.4 eV is ascribed as pyrrole N, 401.58 eV is quaternary N, 402.1 eV to = NH, and 403 eV is associated with NO. In the case of the high-resolution spectrum of O 1s, upon deconvolution (Figure 8E), six types of bonds are obtained, assigned as: 530.92 eV related to molecular oxygen adsorbed on the material, 531.55 eV associated with C-OH bond, 533.3 eV to CO type bonds/C=O, 534.2 eV associated with SO_4_, 534 eV attached to ester or C=NO type bonds, and 535 eV to interstitial water.

**FIGURE 9:**
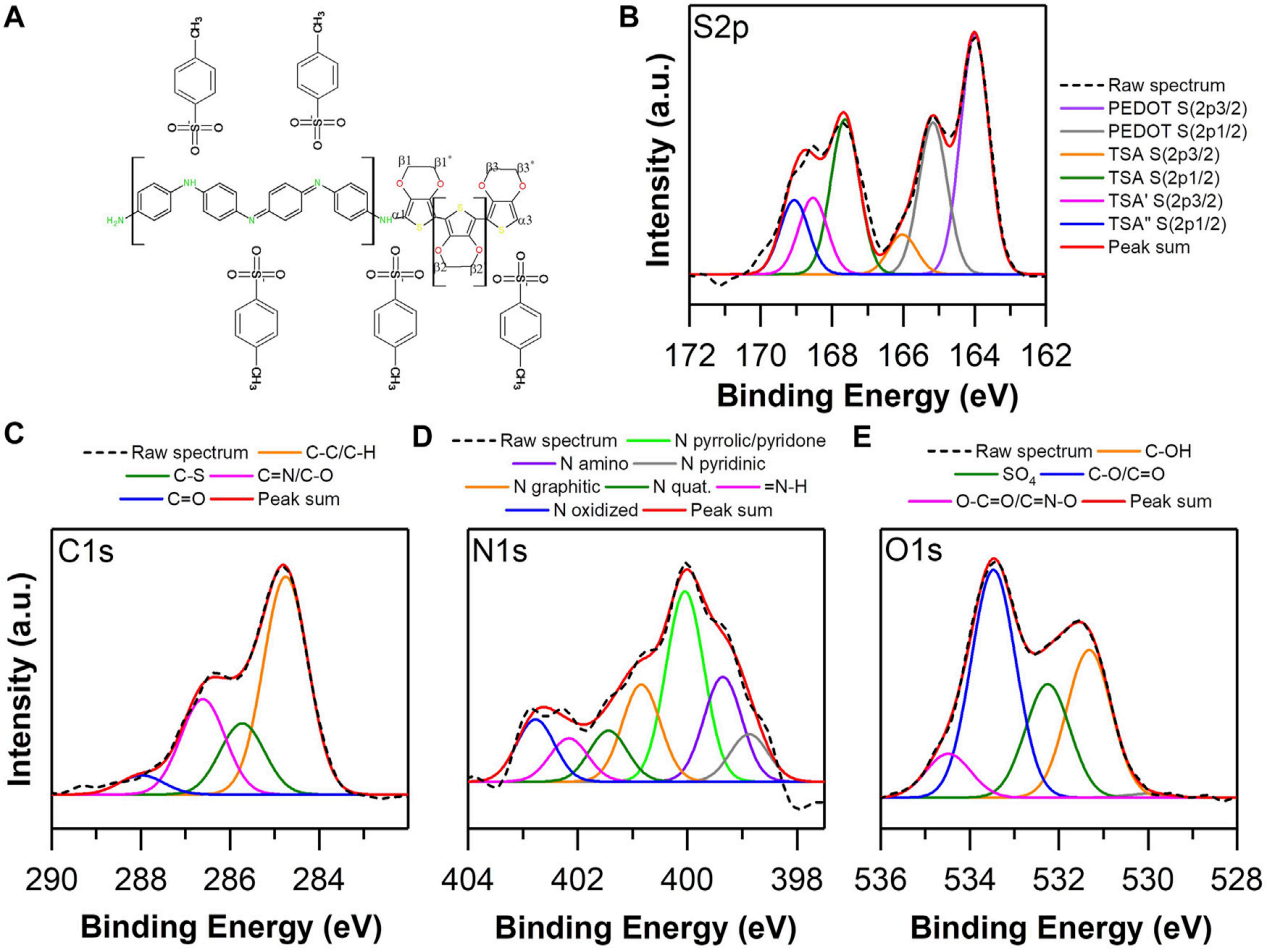
**(A)** Proposed structure of EDOT-ANI TSA Copolymer. High Resolution XPS Spectra of EDOT-ANI 0.7–0.3 TSA: **(B)** S 2p, **(C)** C 1s, **(D)** N 1s and **(E)** O 1s.

The spectral analysis by XPS reveals that the copolymer EDOT-PY 0.7–0.3 differs from the spectra obtained for PEDOT and PPY, which seems to indicate the appearance of pyrrole-EDOT units. The copolymerization process favors the conjugation and doping of the structure with TSA and shares spectral similarities in relation to the PEDOT in terms of the EDOT: TSA doping ratio, which allows us to infer that the electronic structure favors the conduction of current in a similar or even superior way than the PEDOT. Figure 9 presents the high-resolution S, N, C, and O spectra of the EDOT-ANI 0.7–0.3: TSA copolymer. The high-resolution S 2p spectrum (Figure 9B) shows six types of bonds associated with it: 163.8 and 165.2 eV ascribed to the thiophene ring (Fabretto et al., 2009; Wang M. et al., 2016), 166, 167.6, 168.5, and 169 eV are assigned to the S atom of the sulfone of the TSA. The bond at 169 eV does not appear in homopolymers or the EDOT-PY copolymer; this indicates a different interaction between the molecular segments of aniline with TSA, generating a displacement of the binding energy of the sulfone that interacts in these regions of the copolymer. The relationship between the binding energy areas of the S species between thiophene and sulfonic bonds does not correspond to the total doping of the structure; only 70% of the molar composition of the copolymer is based on EDOT. The relationship is close to 1.5:1 EDOT: TSA, being higher than the ratio of PEDOT: TSA and EDOT-PY: TSA, which corroborates an intense interaction between the EDOT-ANI copolymer. TSA is the first experimental evidence on this phenomenon according to our knowledge and could explain certain specific electrical properties that have been reported in the literature for this copolymer, ergo an outstanding electrochemical and electrical performance. The spectrum of C 1s (Figure 9C) exhibits chemical bonds assigned as: 284.5 eV to C sp^3^, 285.7 eV to CS, 286.7 eV to carbonyl/C=N, and 288.2 eV to carboxylate or ester. On the other hand, the high-resolution spectrum of N 1s after deconvolution (Figure 9D) allows identifying seven nitrogen bonds in: 398.8 eV associated with pyridine N, 399.3 eV to pyridyl-type N, 400 eV is ascribed as pyrrolic N, 401 and 401.9 eV are related to quaternary N, 402 eV to = NH, and 402.82 eV is associated with NO. In the high-resolution spectrum of the deconvolved O 1s (Figure 9E), four bonds are distinguished: 530.92 eV related to molecular oxygen adsorbed on the material, 531.34 eV associated with C-OH bond, 532.2 eV related to SO_4_, 533.3 eV type CO bonds/C=O, 534 eV attached to ester or C=NO type bonds, and 535 eV to interstitial H_2_O. The information gathered by the XPS analysis of the homopolymers and copolymers shows chemical differences in their chain structure as well as variations in the degree of doping and oxidation, clearly differing the spectra of the copolymers from the spectra of homopolymers, confirming the formation of the copolymers. Based on EDOT, PY, and ANI, it was observed that the differences in the chemical structure condition change in the dopant concentration, which allows us to argue that the interaction between the counterion and the different copolymers will give rise to different electrochemical and electrical properties.

### Analysis of Particle Size, Z-Potential, and Electrical Conductivity

**FIGURE 10:**
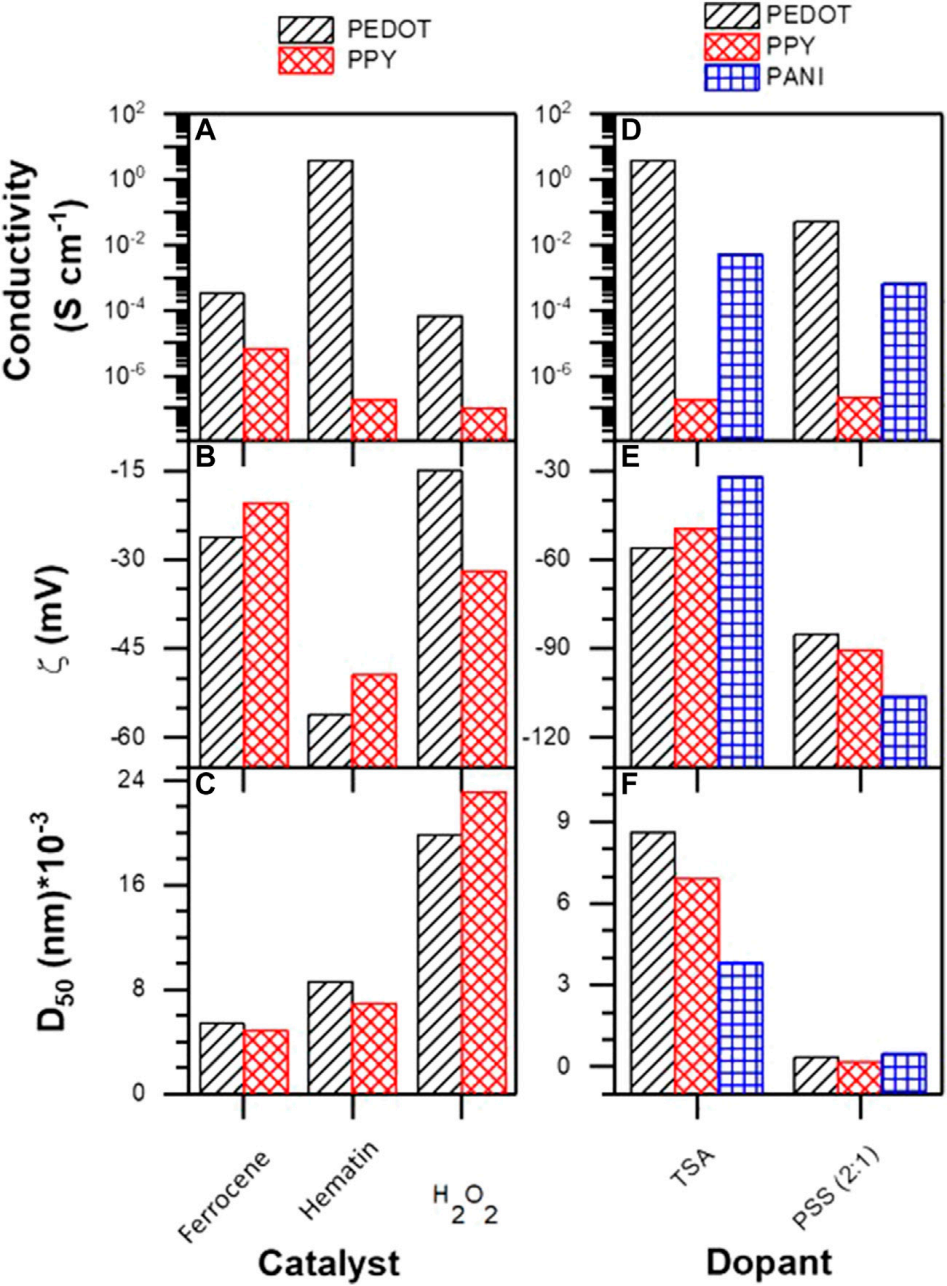
**(A)** Conductivity, **(B)** Z-potential, **(C)** average particle size of PEDOT and PPY doped with TSA, **(D)** conductivity, **(E)** Z-potential, and **(F)** average particle size of PEDOT, PPY, and PANI doped with TSA and PSS.

This section analyzes the effect of the catalyst, the molar fraction of the monomers, and the type of dopant on the particle size, the Z-potential, and the electrical conductivity of homopolymers and copolymers. Such results are essential to determine the products with improved intrinsic properties for the development of inks, deposits, coatings, and electrospinning for use in electrophysiological applications. Figure 10 shows the results obtained for particle size, conductivity, and Z-potential of PEDOT and PPY synthesized using Ferrocene and Hematin as catalysts. In addition, a blank without catalyst was measured. The conductivity of PEDOT increases up to 0.5–1 S/cm when Hematin is used as a catalyst and falls about 5 orders of magnitude with Ferrocene. Due the hematin kinetic data obtained from our team previously, the most probable way of increasing the conductivity of polymers and copolymers through hematin lies in the efficiency to generate radicals that triggered the polymerization and eventually this results in an advantage to obtain a highly ordered polymer backbone, which represents conductive regions (Tierrablanca et al., 2010). Similar results are obtained with the blank without catalyst using only hydrogen peroxide whose conductivity is very low. The conductivity of PPY in all conditions is minimal, between 10^–5^ and 10^–7^ S/cm; the use of hematin results in even lower conductivities. Regarding particle size, the results show a clear trend for both polymers depending on the catalyst: Ferrocene < Hematin < H_2_O_2_. Ferrocene generates smaller particles in the order of 5 microns, hematin between 6 and 9 microns, and the blank without catalyst 20–25 microns. The explanation for the effect of catalysts in reducing particle size lies in the fact that they promote electrostatic impediment in the ionic atmosphere that surrounds the growing particles, which does not favor the attractive interaction by Van der Waals forces. According to the Derjaguin-Landau-Verwey-Overbeek (DLVO) model (Ohshima, 2014), it is an adequate argument to explain the decrease in size depending on the type of catalyst (Supplementary Scheme S2).

Z-potential measurements were carried out at room temperature using deionized water. The Z- potential (ξ) is one of the most important parameters to know the dispersibility and stability of colloids in certain solvent and electrolytic conditions. This parameter corresponds to the electrostatic potential existing in the region between the Stern layer and the beginning of the Gouy-Chapman layer. As the Stern layer becomes not very compact and very extensive, it can almost be considered equal to the surface potential of the colloid (Hermanson, 2013). Thus, despite not being able to measure the surface potential directly, the potential Z is a good indicator under certain conditions. According to Figure 10C, in the absence of catalyst the Z-potentials of PEDOT and PPY are −15 and −32 mV, and with ferrocene they are −26 and −20 mV. The particles of these polymers are unstable since it has been described that above −30 mV the net energy of interaction becomes more electro-attractive and leads to coalescence and sedimentation of the material. On the other hand, PEDOT and PPY obtained using Hematin as a catalyst exhibit −56 and −49 mV respectively, which indicates greater doping with TSA. Together, the three tests show that PEDOT synthesized in the presence of Hematin using TSA as dopant is the material with the most adequate conductivity and stability.

The results of conductivity, Z-potential, and particle size of the homopolymers PANI, PPY, and PEDOT biomimetically synthesized using Hematin as a catalyst and using two different dopants, TSA and PSS, are shown in Figures 10D–F. Regarding the particle size, when the polymers are doped with TSA the sizes are between 1 and 10 microns, while when doping with PSS the decrease in particle size is dramatic, reaching dimensions between 200 and 800 nm. This result clearly indicates that the polyanion has a significant effect on the ionic atmosphere of the growing nuclei, favoring net repulsive energies that give rise to nanometric particles (Hermanson, 2013). Regarding the electrical conductivity of polymers, a decrease in this is observed when doping with PSS instead of TSA; this has been reported by Stöcker et al. (2012) and is typically because of the disorder that the polyanion exerts on the semicrystalline structure of particles, increasing the degree of amorphousness and affecting the interchain charge jump. It should be noted that PEDOT has the highest electrical conductivity, then the PANI and lowest the PPY. This indicates that the degree of disorder and defects in chains changes dramatically between one monomer and another. The Z-potential values found for PEDOT, PPY, and PANI doped with TSA (Figure 10E) were −56, −49, and −30 mV respectively. This indicates that the PEDOT is the one with the highest net repulsion energy of the three materials. Curiously the PANI has the lowest Z-potential, probably due to the presence of positively charged nitrogen that are not doped, which would coincide with quinoid forms of the chain that would give the colloid a more electropositive character and, therefore, make it less stable (Arnold, 1995). However, when doping with PSS, the—potential of the PEDOT, PPY, and PANI decreases to −85, −90, and −106 mV respectively. This effect is since the PSS (potential Z = −76 mV) induces a change in the electrostatic repulsions between colloids (Hermanson, 2013), which makes them more stable. This was corroborated by increasing the amount of PSS from 2:1 to 1:1 and 1:2. In the case of PEDOT, potential reductions of -96 (1:1) and -102 mV (1:2) were observed (Supplementary Figure S10), which indicates that the colloids are very stable in suspension. Supplementary Table S2 shows the reaction yields of the EDOT-PY copolymers doped with TSA. It is observed that there are no significant variations between ferrocene and hematin; however, the trend shows that the yield increases from 75% to 87% approximately as the mole fraction of EDOT decreases.

**FIGURE 11:**
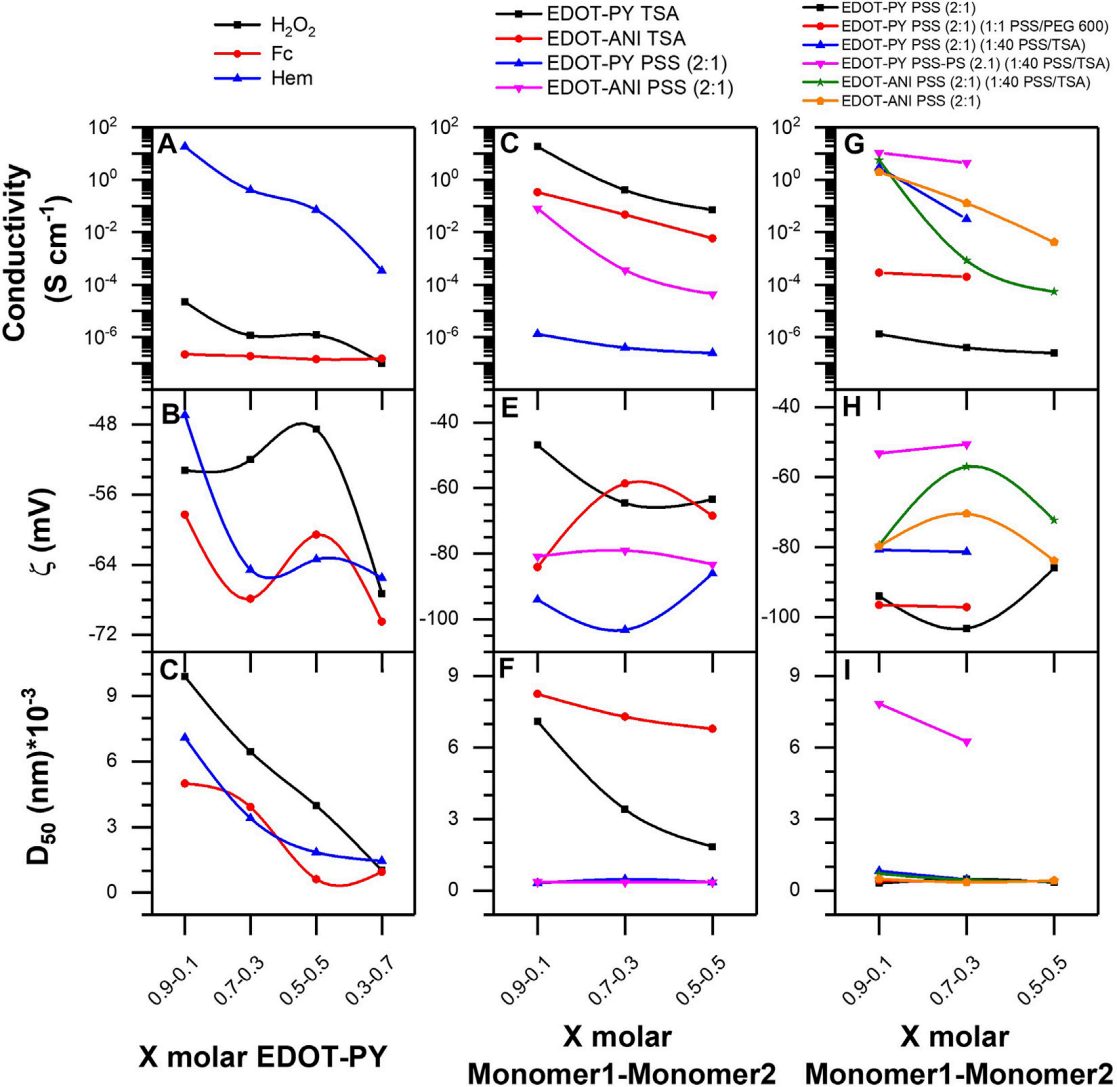
**(A)** Conductivity, **(B)** Z-potential, and **(C)** average particle of EDOT-PY doped with TSA. **(D)** Conductivity, **(E)** Z-potential, and **(F)** average particle of EDOT-PY and EDOT-ANI doped with TSA and PSS. **(G)** Conductivity, **(H)** Z-potential, and **(I)** average particle of EDOT-PY and EDOT-ANI doped with PSS.

On the other hand, when analyzing the changes in the average particle size of the EDOT-PY copolymers depending on the mole fraction of each monomer (Figure 11A–C), a decreasing trend in particle size is observed as the EDOT mole fraction decreases, regardless of the catalyst used. Concerning the Z-potential, the EDOT-PY copolymers have values lower than −45 mV; this indicates that the doping of the copolymers is more efficient than that of the homopolymers. A trend of decrease of the Z-potential is observed as the fraction of EDOT decreases. Although the catalyst does not seem to have a significant effect on the Z-potential, the electrical conductivity does exhibit a behavior that is dependent on the molar fraction of monomer and the type of catalyst. As the molar fraction of EDOT decreases, the resistance of the material increases significantly, causing a significant decrease in conductivity. It should be noted that ferrocene gives rise to semiconductor copolymers (between 10^−4^–10^–7^ S/cm) while hematin gives rise to conductive copolymers with conductivity between 12 and 10^–2^ S/cm. This result is relevant since it shows that, depending on the type of catalyst, the copolymer conductivity can be modified by orders of magnitude. In the same way, by increasing or decreasing the molar fraction of EDOT, the conduction energy gap of the polymers can also be modified.

The results of conductivity, Z-potential, and particle size of EDOT-ANI and EDOT-PY doped with TSA vs. PSS (2:1) obtained using Hematin as a catalyst are presented in Figure 11D–F. Regarding electrical conductivity, the most conductive copolymer was EDOT-PY doped with TSA (1 S/cm of PEDOT versus 12 S/cm of EDOT-PY/TSA 0.9–0.1). However, the doping with PSS was the least conductive, which indicates that the type of dopant significantly affects the conductivity of this copolymer. On the other hand, the conductivity of the EDOT-ANI copolymers doped with TSA is between 0.8 and 0.01 S/cm. The trend is the same as in the case of EDOT-PY; as the EDOT concentration decreases, the conductivity decreases. In the case of EDOT-ANI doping with PSS, conductivities between 0.4 and 10–4 S/cm were found. In both copolymers (EDOT-PY and EDOT-ANI) when doped with TSA, the size varied between 3 and 10 microns, which decreases with increasing molar fraction of pyrrole, however, when doped with PSS the size becomes nanometric (between 400 and 300 nm). Regarding the Z-potential, the EDOT-PY/PSS copolymer has the lowest values indicating a great colloidal stability, being that the 0.7–0.3 M ratio was the one that obtained with the lowest value (-104 mV). The trend was not linear for the different molar relationships, finding that the relationship 0.7–0.3 was the most stable both in doping with PSS and doping with TSA. The EDOT-ANI copolymer also does not show a linear trend in the Z-potential, the 0.9–0.1 (−85 mV) relationship being more stable when doped with TSA. The doping and the molar ratio of the copolymers has a significant effect on the Z-potential, average particle size, and electrical conductivity.

## Conclusion

The spectral analysis (XPS, UV-Vis, Raman, FTIR) confirmed the successful synthesis of EDOT-PY and EDOT-ANI copolymers using two biomimetic catalysts (Ferrocene and Hematin). It was found that the effect of the catalyst at the chemical level is not significant and appears to have small contributions at molar ratios greater than 0.7–0.3. The analysis of particle size, Z-potential, and electrical conductivity allowed to correlate the effect of the catalyst, mole fraction, and dopant on these properties. It was shown that the catalyst causes a decrease on the particle size, while the reactions without catalyst generate very large particles (20 µm). Regarding ferrocene and hematin, ferrocene contributed to obtaining smaller particles (5 µm). On the other hand, the molar fraction also exerts a significant effect on the particle size, as the molar fraction of EDOT decreases, both in the EDOT-PY copolymer and in the EDOT-ANI, there is a decrease in particle size. It was found that the use of TSA gives rise to micrometric scores, which tend to be very unstable. PSS and PSS-*b*-PS give rise to nanoparticles between 100 and 800 nm. In addition to this, it was shown that the copolymers’ Z-potential is more stable than the homopolymers’ Z-potential. Therefore, the EDOT-PY and EDOT-ANI copolymers showed excellent Z-potential values with both TSA and PSS. Finally, the electrical conductivity was considerably modified depending on the type of catalyst; hematin gives rise to conductive homopolymers and copolymers when doped with TSA, while ferrocene gives rise to low semiconductive copolymers under the same conditions. The mole fraction affects conductivity importantly, showing that as the EDOT fraction decreases, the conductivity drops drastically for both EDOT-PY and EDOT-ANI. The type of dopant also notably affects conductivity; the best values were obtained by doping with TSA, while the lowest when doping with PSS. In summary, it was determined that the best molar ratios are between 0.9–0.1 and 0.7–0.3 for both copolymers, the most efficient catalyst was hematin, and the best particle size and Z-potential values were obtained with PSS. Given the results discussed above, this research represents a useful guide that allows scientists in fields like bioengineering, tissue engineering, biophysics, and so on to use conducting polymers and copolymers biomimetically obtained, which are free of highly toxic side products that the typical chemical synthesis pathway produces. This work also represents a novel way to control the conductivity, stability, and size of conjugated semiconductive polymers nanoparticles and microparticles.

## Data Availability

The original contributions presented in the study are included in the article/Supplementary Material, further inquiries can be directed to the corresponding authors.
