# Supplementary material for: A Comparative Study of Biomimetic Synthesis of EDOT-Pyrrole and EDOT-Aniline Copolymers by Peroxidase-like Catalysts: Towards Tunable Semiconductive Organic Materials
Source: Front Chem. 2022 Jun 29;10:915264. doi: 10.3389/fchem.2022.915264 (PMC9278020; doi:10.3389/fchem.2022.915264)
Supplement: Supplementary file 1 [file DataSheet1.ZIP › Figures of SI/Supplementary_Material (002).docx]

Supplementary Material

# Supplementary Data

# Table S1. Homopolymer reaction conditions.

| **Monomer**  **(400 mg)** | **Catalyst type** | **TSA 1M (mL)** | **Co-dopant** | **Additives**  **(100 mg)** | **pH** | **H2O2 uL** |
| --- | --- | --- | --- | --- | --- | --- |
| PY | Ferrocene (10 mg) | 20 | - | linoleic acid | 1.5 | 1000 |
| PY | Ferrocene (40 mg) | 20 | - | - | 1.5 | 1000 |
| PY | Ferrocene (10 mg) | 20 | - | - | 2.9 | 1000 |
| PY | ferrocene (10 mg) | 20 | - | - | 4.2 | 1000 |
| PY | ferrocene (10 mg) | - | - | - | 1.5 | 50 |
| PY | ferrocene (10 mg) | - | - | - | 1.5 | 200 |
| PY | ferrocene (10 mg) | - | - | - | 1.5 | 500 |
| PY | Ferrocene (10mg) | 20 | - | - | 1.5 | 700 |
| PY | Ferrocene (10 mg) | - | 20 mL H2SO4 1M | - | 1 | 1000 |
| PY | FeCl3 (800 mg) | - | 20 mL H2SO41M | - | 1 | 1000 |
| PY | FeCl3 (800 mg) | 20 | - | - | 1.5 | 1000 |
| PY | Hematin (10 mg) | 20 | - | DMSO | 1.5 | 1000 |
| PY | - | 20 | - | - | 1.5 | 1000 |
| EDOT | Ferrocene (5 mg) | 20 | - | - | 1.5 | 1000 |
| EDOT | Ferrocene (10 mg) | 20 | - | - | 1.5 | 1000 |
| EDOT | Ferrocene (40 mg) | 20 | - | - | 1.5 | 1000 |
| EDOT | Hematin (10 mg) | 20 | - | DMSO | 1.5 | 1000 |
| EDOT | - | 20 | - | - | 1.5 | 1000 |
| ANI | Ferrocene (10 mg) | 20 | - | - | 1.5 | 1000 |
| ANI | Hematin (10 mg) | 20 | - | DMSO | 1.5 | 1000 |
| ANI | - | 20 | - | - | 1.5 | 1000 |

**Table S2.** Copolymer reaction conditions.

| **Copolymer (molar fraction EDOT/molar fraction Pyrrole)** | **Catalyst** | **Monomer**  **1** | **Monomer**  **2** | **TSA 1M (mL)** | **pH** | **H2O2 uL** | **Reaction Yield (%)** |
| --- | --- | --- | --- | --- | --- | --- | --- |
| EDOT-PY 0.9-0.1 | - | EDOT (395.5 mg) | PY  (20.5 mg) | 20 | 1.5 | 1000 | 17 |
| EDOT-PY 0.7-0.3 | - | EDOT (307.8 mg) | PY  (61.59 mg) | 20 | 1.5 | 1000 | 77 |
| EDOT-PY 0.5-0.5 | - | EDOT (219.85 mg) | PY  (102.67 mg) | 20 | 1.5 | 1000 | 80 |
| EDOT-PY 0.3-0.7 | - | EDOT (136.6 mg) | PY  (143.74 mg) | 20 | 1.5 | 1000 | 85 |
| EDOT-PY 0.9-0.1 | Ferrocene  (10 mg) | EDOT (395.5 mg) | PY  (20.5 mg) | 20 | 1.5 | 1000 | 78 |
| EDOT-PY 0.7-0.3 | Ferrocene  (10 mg) | EDOT  (307.8 mg) | PY  (61.59 mg) | 20 | 1.5 | 1000 | 80 |
| EDOT-PY 0.5-0.5 | Ferrocene (10 mg) | EDOT (219.85 mg) | PY  (102.67 mg) | 20 | 1.5 | 1000 | 85 |
| EDOT-PY 0.3-0.7 | Ferrocene  (10 mg) | EDOT (136.6 mg) | PY  (143.74 mg) | 20 | 1.5 | 1000 | 92 |
| EDOT-PY 0.9-0.1 | Hematin  (10 mg) | EDOT (395.5 mg) | PY  (20.5 mg) | 20 | 1.5 | 1000 | 74.6 |
| EDOT-PY 0.7-0.3 | Hematin  (10 mg) | EDOT  (307.8 mg) | PY  (61.59 mg) | 20 | 1.5 | 1000 | 83 |
| EDOT-PY 0.5-0.5 | Hematin  (10 mg) | EDOT (219.85 mg) | PY  (102.67 mg) | 20 | 1.5 | 1000 | 86 |
| EDOT-PY 0.3-0.7 | Hematin  (10 mg) | EDOT (136.6 mg) | PY  (143.74 mg) | 20 | 1.5 | 1000 | 87 |
| EDOT-ANI 0.9-0.1 | Hematin  (10 mg) | EDOT (395.5 mg) | ANI  (27.9 mg) | 20 | 1.5 | 1000 | 40 |
| EDOT-ANI 0.7-0.3 | Hematin  (10 mg) | EDOT  (307.8 mg) | ANI  (83.79 mg) | 20 | 1.5 | 1000 | 56 |
| EDOT- ANI 0.5-0.5 | Hematin  (10 mg) | EDOT (219.85 mg) | ANI  (139.68 mg) | 20 | 1.5 | 1000 | 68 |

**Table S3.** Reaction conditions of homopolymers doped with PSS and PSS-*b*-PS.

| Sample | Catalyst  (10 mg) | Monomer 1 | R 1a | R 2b | PSS: co-dopant ratio | Ac | pH | H2O2 uL | Dopant post-synthesis |
| --- | --- | --- | --- | --- | --- | --- | --- | --- | --- |
| PPY | Hematin | PY  (400 mg) | 2:1 | - | - | DMSO 100 mg | 1.5 | 1000 | - |
| PEDOT | Hematin | EDOT  (400 mg) | 1:1 | - | - | DMSO 100 mg | 1.5 | 1000 | - |
| PEDOT | Hematin | EDOT  (400 mg) | 2:1 | - | - | DMSO 100 mg | 1.5 | 1000 | - |
| PEDOT | Hematin | EDOT  (400 mg) | 2:1 | - | - | DMSO 100 mg | 1.5 | 1000 | - |
| PEDOT | Hematin | EDOT  (400 mg) | 1:2 | - | - | DMSO 100 mg | 1.5 | 1000 | - |
| PEDOT (200 mg) | - | - | - | - | - | - | - | - | PEG 600 (20 mg) |
| PEDOT | Ferrocene | EDOT  (400 mg) | 2:1 | - | - | - | 1.5 | 1000 | - |
| PEDOT | Hematin | EDOT  (400 mg) | 2:1 | - | 1:1 PSS/PEG 600) | DMSO 100 mg | 1.5 | 1000 | - |
| PEDOT | Hematin | EDOT  (400 mg) | 2:1 | - | 1:2 PSS/PEG 600 | DMSO 100 mg | - | - | - |
| PEDOT (200 mg) | - | - | - | - | - | - | - | - | PEG 600 (20 mg + 10 mg ethylene glycol) |
| PEDOT | Hematin | EDOT  (400 mg) | - | 1:1 | 1:40 PSS/TSA | DMSO 100 mg | - | - | - |
| PANI | Hematin | ANI  (400 mg) | 2:1 | - | 1:40 PSS/TSA | DMSO 100 mg | - | - | - |

aMonomer: PSS ratio, bMonomer: PSS-*b*-PS ratio and cadditives.

**Table S4.** Reaction conditions for PSS and PSS-*b*-PS doped copolymers.

| Sample | Catalyst | Monomer 1 | Monomer 2 | R 1a | R 2b | PSS: co-dopant ratio | Ac | pH | H2O2 uL |
| --- | --- | --- | --- | --- | --- | --- | --- | --- | --- |
| EDOT-PY 0.9-0.1 | Hematin 10 mg | EDOT 395.5 mg | PY 20.5 mg | 2:1 | - | - | DMSO 100 mg | 1.5 | 1000 |
| EDOT-PY 0.7-0.3 | Hematin 10 mg | EDOT 307.8 mg | PY 61.59 mg | 2:1 | - | - | DMSO 100 mg | 1.5 | 1000 |
| EDOT-PY 0.5-0.5 | Hematin 10 mg | EDOT 219.85 mg | PY 102.67 mg | 2:1 | - | - | DMSO 100 mg | 1.5 | 1000 |
| EDOT-PY 0.7-0.3 | Hematin 10 mg | EDOT 307.8 mg | PY 61.59 mg | 1:2 | - | - | DMSO 100 mg | 1.5 | 1000 |
| EDOT-PY 0.9-0.1 | Hematin 10 mg | EDOT 395.5 mg | PY 20.5 mg | 2:1 | - | 1:1 PSS/PEG 600 | DMSO 100 mg | 1.5 | 1000 |
| EDOT-PY 0.7-0.3 | Hematin 10 mg | EDOT 307.8 mg | PY 61.59 mg | 2:1 | - | 1:1 PSS/PEG 600 | DMSO 100 mg | 1.5 | 1000 |
| EDOT-PY 0.9-0.1 | Hematin 10 mg | EDOT 395.5 mg | PY 20.5 mg | 2:1 | - | 1:40 PSS/TSA | DMSO 100 mg | 1.5 | 1000 |
| EDOT-PY 0.7-0.3 | Hematin 10 mg | EDOT 307.8 mg | PY 61.59 mg | 2:1 | - | 1:40 PSS/TSA | DMSO 100 mg | 1.5 | 1000 |
| EDOT-PY 0.9-0.1 | Hematin 10 mg | EDOT 395.5 mg | PY 20.5 mg | - | 2:1 | 1:40 PSS/TSA | DMSO 100 mg | 1.5 | 1000 |
| EDOT-PY 0.7-0.3 | Hematin 10 mg | EDOT 307.8 mg | PY 61.59 mg | - | 2:1 | 1:40 PSS/TSA | DMSO 100 mg | 1.5 | 1000 |
| EDOT-ANI 0.9-0.1 | Hematin 10 mg | EDOT 395.5 mg | ANI 27.9 mg | 2:1 | - | - | DMSO 100 mg | 1.5 | 1000 |
| EDOT-ANI 0.7-0.3 | Hematin 10 mg | EDOT 307.8 mg | ANI 83.79 mg | 2:1 | - | - | DMSO 100 mg | 1.5 | 1000 |
| EDOT- ANI 5-0.5 | Hematin 10 mg | EDOT 219.85 mg | ANI 139.68 mg | 2:1 | - | - | DMSO 100 mg | 1.5 | 1000 |
| EDOT-ANI 0.7-0.3 | Hematin 10 mg | EDOT 307.8 mg | ANI 83.79 mg | 2:1 | - | 1:1 PSS/PEG 600 | DMSO 100 mg | 1.5 | 1000 |
| EDOT-ANI 0.9-0.1 | Hematin 10 mg | EDOT 395.5 mg | ANI 27.9 mg | 2:1 | - | 1:40 PSS/TSA | DMSO 100 mg | 1.5 | 1000 |
| EDOT-ANI 0.7-0.3 | Hematin 10 mg | EDOT 307.8 mg | ANI 83.79 mg | 2:1 | - | 1:40 PSS/TSA | DMSO 100 mg | 1.5 | 1000 |
| EDOT- ANI 0.5-0.5 | Hematin 10 mg | EDOT 219.85 mg | ANI 139.68 mg | 2:1 | - | 1:40 PSS/TSA | DMSO 100 mg | 1.5 | 1000 |
| EDOT-ANI 0.7-0.3 | Hematin 10 mg | EDOT 307.8 mg | ANI 83.79 mg | - | 1:1 | - | DMSO 100 mg | 1.5 | 1000 |
| EDOT-ANI 0.7-0.3 | Hematin 10 mg | EDOT 307.8 mg | ANI 83.79 mg | 1:1 | - | - | DMSO 100 mg | 1.5 | 1000 |
| EDOT-ANI 0.9-0.1 | Hematin 10 mg | EDOT 395.5 mg | ANI 27.9 mg | 2:1 | - | - | DMSO 100 mg | 1.5 | 1000 |
| EDOT-ANI 0.7-0.3 | Hematin 10 mg | EDOT 307.8 mg | ANI 83.79 mg | 2:1 | - | - | DMSO 100 mg | 1.5 | 1000 |
| EDOT- ANI 0.5-0.5 | Hematin 10 mg | EDOT 219.85 mg | ANI 139.68 mg | 2:1 | - | - | DMSO 100 mg | 1.5 | 1000 |
| EDOT-ANI 0.7-0.3 | Hematin 10 mg | EDOT 307.8 mg | ANI 83.79 mg | - | 2:1 | - | DMSO 100 mg | 1.5 | 1000 |

aMonomer: PSS ratio, bMonomer: PSS-*b*-PS ratio and cadditives.

**Scheme S1. (A)** Proposed EDOT-ANI copolymer structure and **(B)** proposed EDOT-PY copolymer structure.



**Figure S1.** UV-vis spectra of PANI.

**Polyanion doped copolymers UV-vis analysis.**

**Figure S2** shows different UV-vis spectra of PEDOT, PPY and EDOT-PY copolymers doped with PSS (Mn ~68000 g/mol, D50=3 nm), PSS-*b*-PS (PSS136-*b*-PS620, D50=100 nm and PSDLS=1.2) and *p*-toluene sulfonic acid (TSA) in different ratios. In all cases, a monomer:PSS ratio of 2:1 by weight was used. In the PEDOT-PSS spectrum (**Figure S2**), a broad band is observed that starts at approximately 600 nm and extends towards the NIR, with at least three distinguishable absorption maxima at 820, 920 and 1090 nm, these transitions are associated with polarons and bipolarons located throughout the chain, whose existence can be visualized as interband sublevels of p-type carriers (Santos et al., 2007; Funda et al., 2016). Regarding the PPY-PSS spectrum, a transition centered at 450 nm is observed that can be interpreted as the jump from HOMO to antibonding orbitals produced by the loss of neutrality of the chain and its evolution towards polaronic charge carriers (Kępas et al., 2007), this transition decays towards the NIR which implies changes in conjugation through the chain, probably caused by a decrease in the degree of doping since the amount of polyanion is small compared to the amount of monomer, a faint transition is also observed at 1014 nm confirming the weak character of bipolaronic or bicationic carriers in the chain and is most likely associated with loss of electrical conductivity. In the case of the EDOT-PY copolymers doped with PSS, the spectrum of the 0.9-0.1 molar ratio shows four absorptions centered at 400, 450, 730 and 1000 nm, the first is associated with the polaron of the pyrrolic segment (Kępas et al., 2007), the second is the transition obtained by changing the neutral character of the thiophenic segment towards p-type carriers in the chain (Rubio et al., 2001), with respect to the sub gap at 700 nm, can undoubtedly confirm what was discussed in the uv-vis monitoring of the reaction of said copolymer, and that such electronic transition is associated with the polaron located in the molecular segment formed by EDOT-PY, while the signal at 1000 nm is a peak of bipolaronic character of the EDOT (Garreau et al., 2001a) molecular segment, in the case of the 0.7-0.3 ratio an intense absorption is observed with a maximum at 400 nm that coincides with the previous description, however, the signal at 450 nm is not present, which differs from the preliminary observation in the reaction monitoring in the absence of PSS, whose only explanation is lies in an extreme redshift caused by the efficient doping of the polyanion on the longest thiophenic segments (Rubio et al., 2001), it is probably a matter of statistically possible collisions in the reaction where the number of EDOT molecules decreases, giving rise to a lower number of events which produce short segments alternating with short pyrrole segments, giving preference to forming longer PEDOT segments alternating with short oligopyrrole segments. In order to confirm whether the effect on the variation of the electronic transitions is a direct effect of the doping or if it is an effect of the way the reaction itself proceeds in the presence of polyanionic templates, TSA was introduced as PSS co-dopant, in the spectrum of the EDOT-PY 0.9-0.1 copolymer exactly the same electronic transitions are observed, surprisingly when obtaining the spectrum of the EDOT-PY 0.7-0.3 copolymer it reproduces in a very similar way the shape of the spectrum only doped with PSS, this conclusively demonstrates that variation on interband sublevels depends directly from the molar ratio and the presence of a polyanionic template, and does not have an influence directly attributable to the high doping of the chain, this finding is remarkable since the very variation of the molar ratio of the monomers in the presence of a polyanionic dopant does have an effect on the electronic structure of the skeleton formed. Finally, this experiment was reproduced under the same doping conditions used in the PSS/TSA but changing the polyanion for nanoparticles of a PSS-PS block copolymer, which is the first time it has been used to dope semiconductor polymers, since there is only a report of a random copolymer recently published by Im et al. (Im et al., 2019), but its effect during the synthesis on the electronic structure is not analyzed. In the spectrum of the EDOT-PY 0.7-0.3 copolymer, but using PSS-PSS/TSA, it is observed that the pattern of the spectrum looked in the previous ones is reproduced, this confirms the effect that the polyanionic template has on the electronic structure of the copolymers, since that it is dependent on the length of the polyanionic segment and serves as a template to favor the growth of preferentially ordered thiophenic segments that gives rise to electronic delocalization length, which allows the appearance of sublevels of lower energy (Wang et al., 2015), this was corroborated

by conductivity measurements.

**Figure S2.** UV-vis spectra of EDOT-PY homopolymers and copolymers doped with PSS and other co-dopants

**Figure S3** shows the spectra of EDOT-ANI doped with PSS and PSS/TSA, in all cases the monomer:PSS ratio was 2:1 by weight, and 1:40 PSS/TSA, the PANI-PSS spectrum reveals five identifiable transitions at 400, 450, 560, 820 and 1020 nm, the first two are associated with HOMO-LUMO transitions in degenerate orbitals by cation-type charge localization in quinoid segments and is consistent with theoretical results reported by Zhekova et al. (Zhekova et al., 2007), the 560 nm signal could be considered as an electronic transition type HOMO-antibonding molecular orbital (π-π*) of the BQB form of PANI (Xia et al., 1994), the signals at 820 and 1020 nm can be associated with bipolarons or bications confined with energies located between 1.54 and 1.22 eV (Kalantari Fotooh et al., 2018), indicates that it is generally the emerald phase of polyaniline, according to the reported theoretical calculations, PANI is much more complex in terms of electronic transitions with respect to PPY or PEDOT, since the sublevels occur between intramolecular or even intermolecular π-π* transitions (Kalantari Fotooh et al., 2018), which gives rise to a large number of energetic sublevels, for which the loss of conjugation caused by any factor (pH, synthesis conditions, etc.) has as a consequence a significant decrease in electrical conductivity, since charge mobility depends largely on perfectly ordered chains. The UV-vis spectrum of the EDOT-ANI 0.9-0.1 copolymer doped with PSS exhibits three important transitions, the first at 400 nm is of the π-π* type, the second whose absorption maximum is at 790 nm is associated with bipolaron-type transitions or polaron but of the molecular segments of EDOT (Garreau et al., 2001b) and EDOT-ANI, it is prominent but the position indicates segments with less conjugation with respect to PEDOT, the last electronic transition centers at 1012 nm due to its intensity it is ascribed to bications or biradicals confined within the polythiophenic-type molecular segments (Garreau et al., 2001b). The 0.7-0.3 copolymer shows a redshift of the π-π* transition of higher frequency due to the increase of aniline in the skeleton, this bathochromic shift is observed in the second transition that passes from 790 to 800 nm, indicating an increase in the length of quinoid segments (Xia et al., 1994).. The third transition maintained the position since it has a greater electronic contribution from the PEDOT segments, however, a shoulder appears at 1080 nm that may be a sublevel related to the BQB segments of the polyaniline or Q-Ty-Q of the part of the copolymer, it is difficult to define these transitions with certainty due to the complexity of the copolymer (Kamran et al., 2015). Finally, in the copolymer 0.5-0.5 it is observed that there is no change in the positions with respect to the copolymer 0.7-0.3, however between the first and second transition an absorption tail extends due to the appearance of small overlapping electronic contributions probably to polaronic bands of the copolymers and π-π* contributions of the longer polyaniline segments than those found in the two previous copolymers, because of the increase in monomer concentration. Finally, it is worth mentioning that the same copolymers synthesized in the presence of TSA as co-dopant accurately reproduce the results described above, which confirms our assumption that the electronic contributions of the dopant ions are small and do not have a macromolecular effect as observed by the molar ratio between monomers, which is consistent with the EDOT-PY copolymers, however the charge distortions on p-type carriers should be modified in terms of electrical conductivity properties, which

is discussed in the section on electrical measurements and the effect of dopants on it.

**Figure S3.** UV-vis spectra of EDOT-ANI homopolymers and copolymers doped with PSS and other co-dopants.

**Table S5.** Summarizing of several IR vibrational modes on PEDOT: PSS, PPY: PSS and EDOT-PY: PSS copolymers at different molar fractions.

|  |  |  |  | **EDOT-PY PSS (2:1)** |  | **EDOT-PY PSS (2:1) (1:40 PSS /TSA)** |  | **EDOT-PY PSS-PS (2:1) (1:40 PSS-PS /TSA)** |
| --- | --- | --- | --- | --- | --- | --- | --- | --- |
| **Assignation** | **PEDOT PSS** | **PPY PSS** | **0.9-0.1** | **0.7-0.3** | **0.9-0.1** | **0.7-0.3** | **0.9-0.1** | **0.7-0.3** |
| **ν(C=O)** | - | - | - | 1726 | - | - | - | - |
| **ν(C=N)** | - | 1709 | - | - | - | - | - | - |
| **ν(C=N), δ(NH), ν(-OH)** | 1631 | 1626 | 1637 | 1637 | - | - | - | - |
| **ν(C=C) asym, heterocyclic ring quinoid (p doped)** | 1599 | 1600 | - | - | - | - | - | - |
| **ν(C=C) asym benzenoid heterocycle** | 1545 | - | - | - | 1511 | - | 1510 | 1510 |
| **ν(C=C) sym, benzenoid heterocycle (n doped)** | 1493 | 1493 | 1484 | 1484 | 1479 | 1480 | 1479 | 1479 |
| **ν(C=C) sym, heterocyclic ring quinoide (p doped)** | 1477 | 1443 | 1444 | 1444 | - | - | 1443 | 1443 |
| **ν(C-C ) asym heterocycle** | 1445 | - | - | - | - | - | - | - |
| **ν(SO3) asym TSA** | 1409 | 1407 | 1393 | 1393 | 1390 | - | 1392 | 1392 |
| **ν(C-C) sym, ν(C-O-C) EDOT Oxyethylene ring** | 1352 | - | 1362 | 1362 | - | 1357 | - | - |
| **δ(C-N), ν(N-H)** | - | 1300 | - | - | 1306 | - | 1307 | 1307 |
| **δ(C-C) on plane ring** | 1209 | 1209 | 1221 | 1221 | - | - | - | - |
| **ν(SO3)** | 1172 | 1172 | 1178 | 1178 | 1178 | 1178 | 1180 | 1180 |
| **δ(C-O-C)** | 1126 | 1126 | 1131 | 1131 | 1133 | 1133 | 1140 | 1140 |
| **δ(S-O-R) TSA** | 1077 | 1077 | 1083 | 1083 | 1081 | 1081 | 1084 | 1084 |
| **δ(C-C) heterocyclic ring** | 1033 | 1033 | 1038 | 1038 | 1050 | 1050 | 1050 | 1050 |
| **δ(C-H) out plane ring** | 1006 | 1006 | 1011 | 1011 | 1007 | 1007 | 1007 | 1007 |
| **δ(C-H) wagging** | 976 | 948 | 992 | 992 | 967 | 967 | 974 | 974 |
| **δ(C-S) thiophenic ring** | 923 | - | 941 | 941 | - | - | - | - |
| **δ(C-C) heterocyclic ring** | 832 | 835 | 841 | 841 | - | - | - | - |

**Table S6.** Band assignment of FTIR spectra of PEDOT, PANI and EDOT-ANI doped with PSS and other co-dopants.

|  | **Bands (cm-1)** |  |  |  |  | **EDOT-PY Fc** |  |  |  | **EDOT-PY Hem** |  |  |
| --- | --- | --- | --- | --- | --- | --- | --- | --- | --- | --- | --- | --- |
| **Assignation** | **PPY Hem** | **PPY Fc** | **PEDOT Hem** | **PEDOT Fc** | **0.9-0.1** | **0.7-0.3** | **0.5-0.5** | **0.3-0.7** | **0.9-0.1** | **0.7-0.3** | **0.5-0.5** | **0.3-0.7** |
| **ν(C=O)** | - | - | - | - | 1742 | 1721 | 1721 | 1721 | - | - | - | - |
| **ν(C=N)** | 1708 | - | - | - | 1711 | - | - | - | - | 1708 | 1708 | 1708 |
| **ν(C=N), δ(NH), ν (-OH water)** | - | - | - | - | - | 1623 | 1623 | 1623 | - | - | - | - |
| **ν(C=C) asym, heterocyclic ring quinoide (p doped)** | 1566 | 1528 | 1539 | - | 1532 | 1561 | 1561 | 1561 | - | 1555 | 1555 | 1555 |
| **ν(C=C) asym benzenoid heterocycle** | 1491 | 1494 | 1518 | - | 1473 | 1483 | 1483 | 1483 | 1474 | 1484 | 1484 | 1484 |
| **ν(C=C) sym, benzenoid heterocycle (n doped)** | 1458 | - | 1476 | 1470 | 1447 | 1449 | 1449 | 1449 | - | - | - | - |
| **ν(C=C) sym, heterocyclic ring quinoide (p doped)** | 1420 | 1410 | 1438 | - | - | - | - | - | - | - | - | - |
| **ν(C-C) asym heterocycle** | 1365 | - | 1393 | 1396 | 1384 | 1401 | 1401 | 1401 | 1384 | - | - | - |
| **ν(SO3) asym TSA** | - | - | - | 1332 | 1333 | 1350 | 1350 | 1350 | - | 1356 | 1356 | 1356 |
| **ν(C-C) sym, ν(C-O-C) EDOT Oxyethylene ring** | - | - | 1305 | - | - | 1295 | 1295 | 1295 | 1306 | 1278 | 1278 | 1278 |
| **δ(C-N), ν(N-H)** | 1211 | 1205 | - | - | - | 1219 | 1219 | 1219 | - | 1216 | 1216 | 1216 |
| **δ(C-C) on plane ring** | 1167 | 1156 | 1178 | 1185 | 1185 | 1150 | 1150 | 1150 | 1131 | - | - | - |
| **ν(SO3)** | 1118 | 1115 | 1131 | 1140 | 1126 | 1127 | 1127 | 1127 | - | 1116 | 1116 | 1116 |
| **δ(C-O-C)** | - | - | - | 1082 | 1086 | 1089 | 1089 | 1089 | 1076 | - | - | - |
| **δ(C-H) on plane ring** | 1031 | - | 1024 | 1027 | 1032 | 1036 | 1036 | 1036 | 1043 | 1030 | 1030 | 1030 |
| **δ(S-O-R) TSA** | 1000 | 1006 | - | - | 1008 | 1012 | 1012 | 1012 | 1005 | 1006 | 1006 | 1006 |
| **δ(C-C) heterocyclic ring** | 969 | 966 | 954 | 977 | 970 | 988 | 988 | 988 | 965 |  |  |  |
| **δ(C-H) out plane ring** | 926 | 932 |  |  | 928 | 947 | 947 | 947 |  | 934 | 934 | 934 |
| **δ(C-H) wagging** | 809 | 813 | 816 | 826 | 836 | 850 | 850 | 850 | 893 | 839 | 839 | 839 |
| **δ(C-S) thiophenic ring** |  |  |  | 752 | 817 | 819 | 819 | 819 | 810 | 809 | 809 | 809 |
| **δ(C-C) heterocyclic ring** | 683 | 681 |  |  | 687 |  |  |  |  | 681 | 681 | 681 |

**TABLA S7.** Band assignment of FTIR spectra of PEDOT, PANI and EDOT-ANI obtained with different catalysts.

|  |  |  | **EDOT-ANI Hem** |  |
| --- | --- | --- | --- | --- |
| **Assignation** | **PEDOT Hem** | **PANI Hem** | **0.9-0.1** | **0.7-0.3** |
| **ν(C=C) asym, heterocyclic quinoide ring (p doped)** | 1539 |  |  |  |
| **ν(C=C) asym benzenoid heterocycle** | 1518 | 1559 | 1535 | 1535 |
| **ν(C=C) sym, benzenoid heterocycle (n doped)** | 1476 | 481 | 1474 | 1474 |
| **ν(C=C) sym, heterocyclic quinoide ring (p doped)** | 1438 | 1438 | 1437 | 1437 |
| **ν(C-C ) asym heterocycle** | 1393 |  | 1385 | 1385 |
| **ν(SO3) asym TSA** |  |  |  |  |
| **ν(C-C) sym, ν(C-O-C) EDOT Oxyethylene ring** | 1305 | 1290 | 1318 | 1318 |
| **δ(C-N), ν(N-H)** |  | 1208 |  |  |
| **δ(C-C) on plane ring** | 1178 |  |  |  |
| **ν(SO2)** | 1131 | 1131 | 1194 | 1194 |
| **δ(C-O-C)** |  | 1104 | 1138 | 1138 |
| **δ(C-H) on plane ring** | 1024 |  | 1124 | 1124 |
| **δ(S-O-R) TSA** |  |  | 1050 | 1050 |
| **δ(C-C) heterocyclic ring** | 954 | 1030 |  |  |
| **δ(C-H) out plane ring** |  | 1003 | 1005 | 1005 |
| **δ(C-H) wagging** | 816 |  | 974 | 974 |
| **δ(C-S) thiophenic ring** |  | 932 | 931 | 931 |
| **δ(C-C) heterocyclic ring** |  | 874 | 835 | 835 |

**FTIR**

The FTIR spectrum in **Figure 4c** shows a 1565 cm-1 signal attributed to C=C stretching in the quinoid ring; a signal at 1590 cm-1, is attributed to the N-H (Trchová and Stejskal, 2011), bending; the signals at 1482 and 1442 cm-1 are attributed to the C=C stretch in the benzenoid ring (Trchová and Stejskal, 2011); the signal at 1375 cm-1 corresponds to the CN stretch in QBQ units; the peak at 1332 cm-1 is ascribed to the CN stretch; the 1285 cm-1 band corresponds to CN stretches of secondary aromatic amine (Trchová and Stejskal, 2011); the peak at 1240 cm-1 is associated with the CN stretch of the BBB unit (Trchová and Stejskal, 2011; Mostafaei and Zolriasatein, 2012); the signal of 1103 cm-1 corresponds to the BNB stretch of the chain (Trchová and Stejskal, 2011); the signal of 1029 cm-1 is associated with S=O stretches resulting from doping with TSA in the sample (Tierrablanca et al., 2010); a small band at 875 cm-1 can detect the deformation outside the CH plane of rings (Trchová and Stejskal, 2011); bands close to 820 cm-1 are characteristics of *p*-ring substitutions; and the band at 781 cm-1 is attributed to deformation out of the C-H plane for 1,2 (ortho) monosubstituted ring (Trchová and Stejskal, 2011).

**Figure S4** shows the FTIR spectra of PEDOT, PPY and EDOT-PY doped with PSS, PSS-PS and TSA in the following ratios: monomer/PSS or PSS-PS (2:1) and PSS or PSS-PS/TSA (1:40). In the case of the PEDOT PSS spectrum, the existing bands corroborate the chemical structure of the polymer according to literature, contributions from the PSS are also observed, specifically from the -SOx group, which indicates that the doping process with PSS was successful. The spectrum of PPY PSS shows characteristic bands observed in the case of PPY doped with TSA, in general terms typical chemical groups of the polymer are observed. When analyzing the bands of the EDOT-PY PSS copolymer (**Table S5**), the formation of the copolymer is corroborated, with respect to the molar composition, similarities are observed in practically identical positions, which confirms the chemical structure of the synthesized copolymer and reproduces what was observed in the case of doping with TSA, the same trend is observed when introducing TSA as a dopant, however the intensity of the bands decreases and the spectral pattern is modified to a certain degree, this probably makes sense when considering the increase in dopant ions, according to Sandoval et al., the change in the doping of the structure causes small variations in intensity and position of some bands, especially at frequencies in the region between 1500-1400 cm-1, and coincides with our experimental observations, even some may disappear, especially those related to neutral segments of the chain, we believe that the change in position of some bands and the modified intensity a of the spectral pattern is directly related to the increase in the degree of doping, such variations recorded in the FTIR spectrum may indirectly indicate the increase in doping in the structure synthesized in the presence of polyanion and an organic acid such as TSA. The EDOT-PY and PSS-PS/TSA spectra agree with what has been described previously and provide guidelines to determine that the PSS or PSS-PS co-doping with TSA modifies the intensity of the bands and slightly their position due to the increase in p-type doping. Another interesting feature of the spectra is that, regardless of the type of dopant, the region between 1200 and 1000 cm-1 is equivalent between the different products, it is not a strange result since it is expected that a structure with a chemical character not dependent on the dopant retain similar chemical functionalities, it confirms that the EDOT-PY copolymers show their own FTIR spectral fingerprint under the investigated synthesis conditions.

FTIR spectra of PANI and EDOT-ANI copolymers with different dopants and synthesis molar ratios are presented in **Figure S5**. In the case of the PANI PSS spectrum, the characteristic bands previously discussed in the case of doping with TSA are observed, likewise the bands related to -SOx groups are perfectly unidentified and confirm doping with PSS, while for EDOT copolymers -ANI PSS we observe the existence of typical bands of both heterocyclic rings (EDOT and Aniline), **Table S8** shows the assignments of the different bands detected, it is worth mentioning that depending on the molar ratio there are position variations in the region between 1500 and 1400 cm-1, which could mean changes in the doping of the chain and in the conjugation of the skeleton, since at lower PEDOT content the bands related to C=C stretching of the ring change and in the case of the 0.5-0.5 copolymer even decrease notably such that they cannot be assigned with precision, on the other hand in the region that includes 1300-1000 cm-1 a high correlation of the modes is observed vibrational s of the functionalities present that indicates equivalence at the structural level. In the case of simultaneous doping with TSA and PSS, it is determined that they present comparable spectral patterns, this is a result that illustrates the direct effect that co-doping has, and its explanation probably lies in a considerable increase in p-type doping of the structure that has as a consequence, homogenize the oxidation state of the chain and give rise to practically identical vibrational modes since they result from equivalent molecular segments, however, a slight decrease in the intensities of the bands between 1500-1400 cm-1 is observed in the 0.5-0.5 copolymer, which is not as drastic as in the case of single doping with PSS, and such an assessment could be relevant to demonstrate the change in the oxidation state of the polymer through

said variation in the bands, and which has been raised previously by Sandoval et *al.*

**Figure S4.** FTIR spectra of homopolymers and copolymers doped with PSS and other co-dopants: PEDOT, PPY and EDOT-PY.

**

Figure S5.** FTIR spectra of homopolymers and copolymers doped with PSS and other co-dopants: PEDOT, PANI and EDOT-ANI.

**X-Ray Diffraction Analysis of Homopolymers and Copolymers Doped with PSS, PSS-PS and TSA**

**Figure S6** shows the PEDOT diffractograms. PPY and EDOT-PY with different doping, in the case of PEDOT-PSS (2:1) the diffractions become wider with respect to what was analyzed with the TSA doping studied above, this is because the PSS being a semicrystalline polymer introduces a higher level of amorphousness to the structure, despite this an extremely intense and narrow peak is observed at 2θ=4.9°, which could indicate a significant increase in the order between adjacent segments on the XY (Wang et al., 2018) plane, in the case of PPY PSS (2:1) diffractions are extremely broad, however a diffraction at approximately 2θ=30° does not appear when only doped with TSA, so it could be associated with PSS chain stacking (Zhou et al., 2015; Bahry et al., 2018; Wang et al., 2018; Kim et al., 2019). When analyzing the diffractograms of homopolymers and copolymers with different dopant: (A) PEDOT, PPY and EDOT-PY doped with PSS or PSS-PSS (2:1) and TSA (1:40 PSS or PSS-PS/TSA), ( B) PEDOT, PANI and EDOT-ANI doped with PSS or PSS-PSS (2:1) and TSA (1:40 PSS or PSS-PS/TSA) show four broadened diffractions, indicating an increase in the disorder of the structure both due to the loss of planarity introduced by the copolymer and the presence of PSS (Anothumakkool et al., 2015), in the case of the polyanion PSS-PS and TSA an interesting effect occurs in the plane associated with the stacking of chains (2θ=26.2°), since it narrows the peak, which gives indications of higher order in the stacking of chains, this could mean an increase in the electrical conductivity of the material, the explanation probably lies in the shape and size of the polyanion nanoparticles that can lead to a higher order, or that serve as nucleation and growth points for crystalline nanodomains. **Figure S7** shows the diffractogram of the PANI PSS (2:1) which presents four broad signals centered at: 2θ=5, 16.7, 28.6 and 41.1° (described in the main document) (Roy et al., 2002; Zhang et al., 2010; Mostafaei and Zolriasatein, 2012), these diffractions are very wide and rounded, which indicates that the PSS introduces a high degree of amorphousness to the structure, with respect to the EDOT-ANI PSS copolymers (2:1) the same four diffractions with Gaussian form are observed, this indicates that the copolymers are highly amorphous, and coincides with that described for the EDOT-PY copolymers. Finally, the EDOT-ANI copolymers co-doped with PSS/TSA show diffractograms like those doped with PSS, in simple terms no significant effect of codoping on the crystal structure is observed, in such a way that the reported effect of the copolymer is reliably verified. PSS in the structure of conjugated polymers by increasing their amorphousness.

**

Figure S6.** Diffractogram of PEDOT, PPY and EDOT-PY copolymers with different molar ratio doped with TSA and using Hematin as catalyst.

**Figure S7.** Diffractogram of PEDOT, PANI and EDOT-Ani copolymers with different molar ratio doped with TSA and using Hematin as catalyst.

**Figure S8.** PEDOT TSA XPS spectrum and element concentration.

**Figure S9.** PPY TSA XPS spectrum and element concentration.


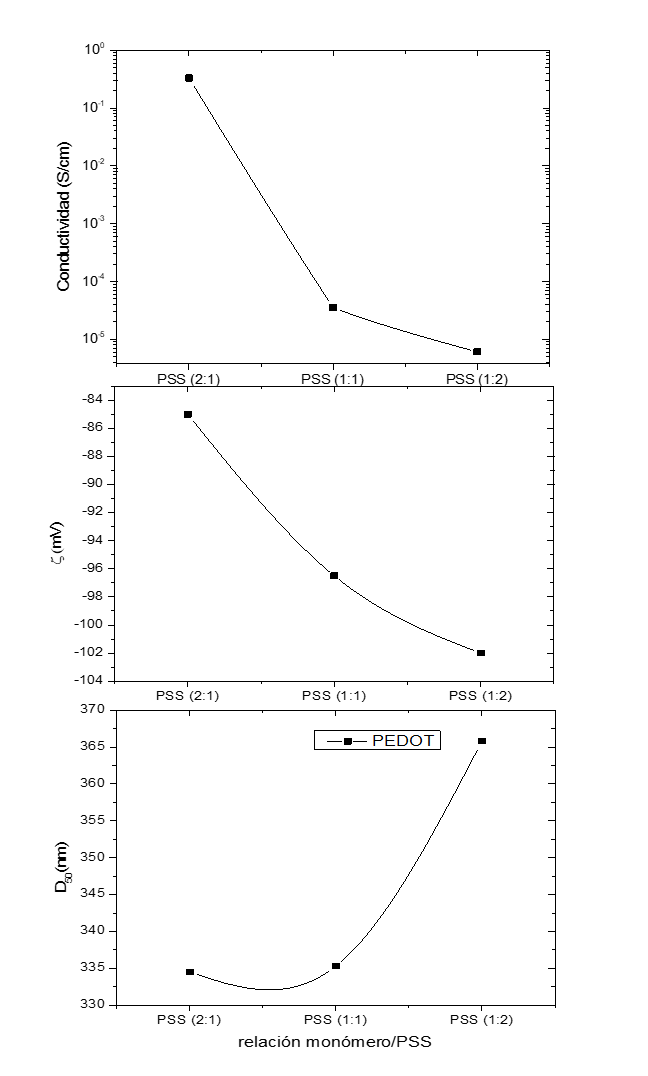


**Figure S10.** Average particle size and Z-potential of PEDOT doped with PSS in different monomer/PSS weight ratios.

**Figure S11.** 1H NMR spectrum of sulfonated styrene in D2O.

**
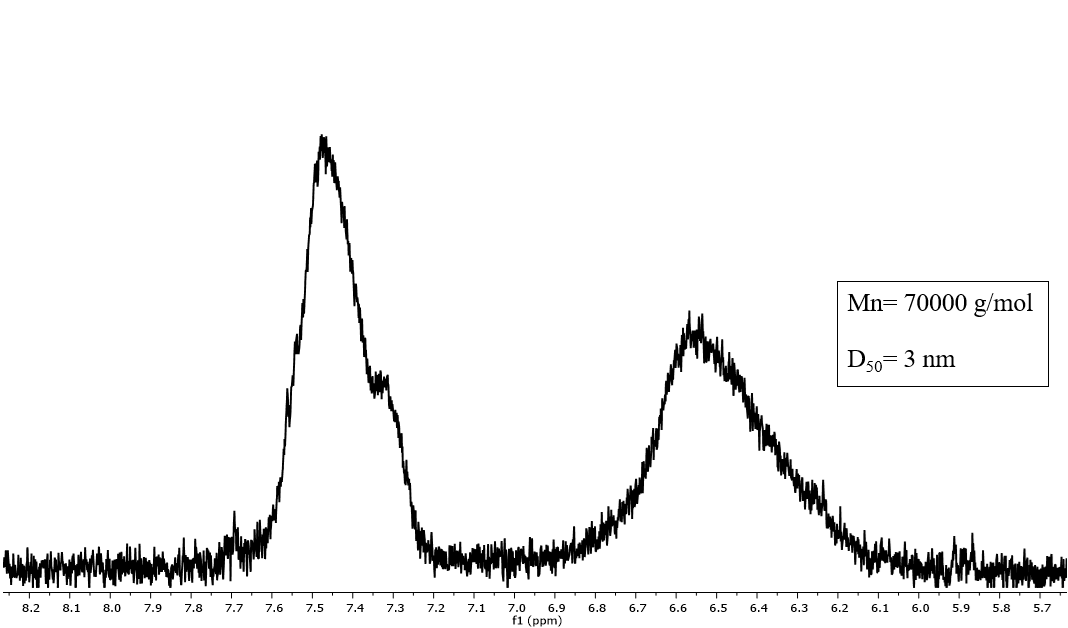
**

**Figure S12.** 1H NMR Spectrum of PSS Mn 70000.

**
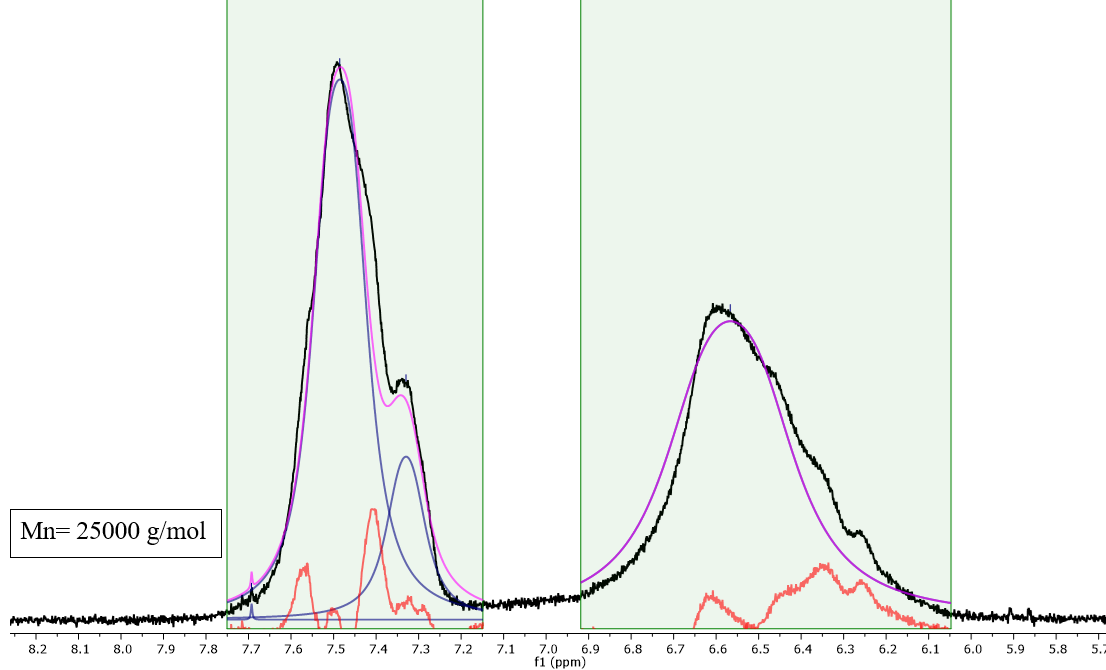
**

**Figure S13.** 1H NMR Spectrum of PSS Macro CTA.

**
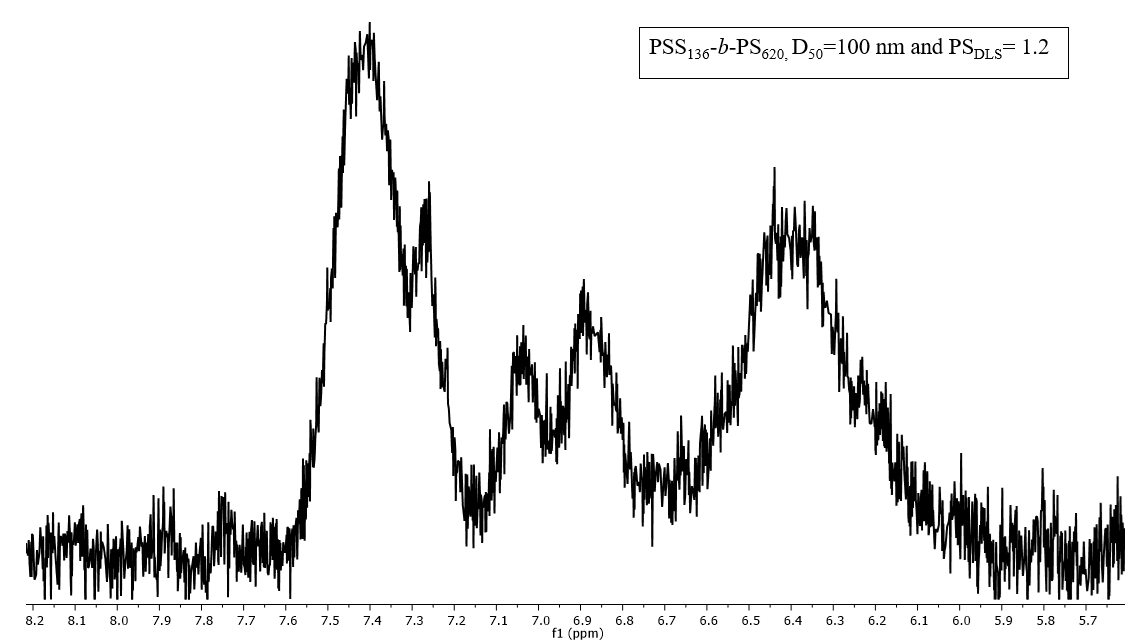
**

**S14.** 1H NMR spectrum of PSS-*b*-PS.

**
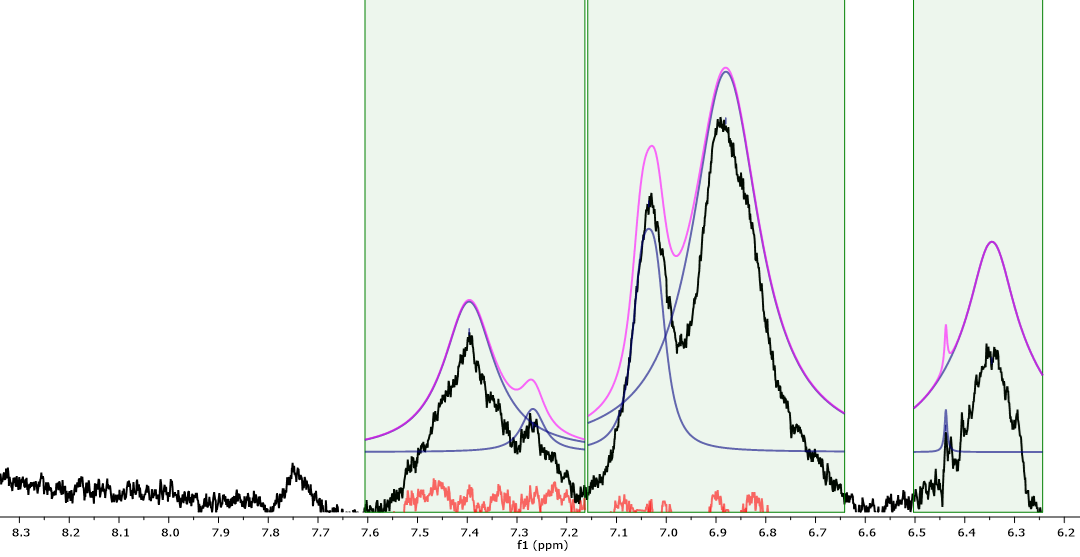
**

**Figure S15.** 1H NMR deconvolved spectrum of PSS-*b*-PS.

**
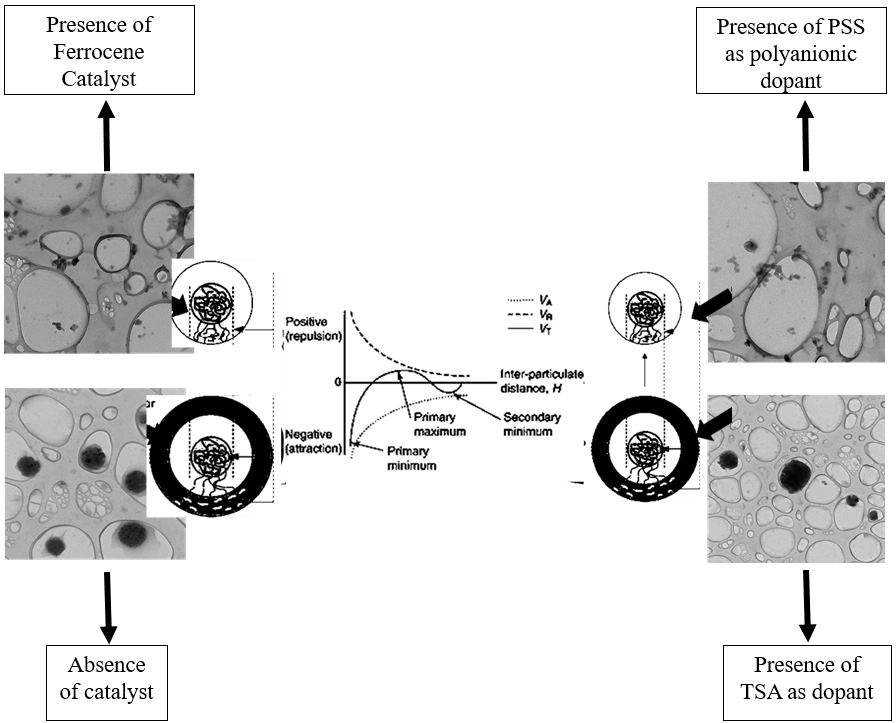
**

**Scheme S2.** According to the DLVO model, it is an adequate argument to explain the decrease in size depending on the type of catalyst (or without catalyst) and kind of dopant.

**References**

Anothumakkool, B., Soni, R., Bhange, S. N., and Kurungot, S. (2015). Novel scalable synthesis of highly conducting and robust PEDOT paper for a high performance flexible solid supercapacitor. *Energy Environ. Sci.* 8, 1339–1347. doi:10.1039/C5EE00142K.

Bahry, T., Cui, Z., Deniset-Besseau, A., Gervais, M., Sollogoub, C., Bui, T.-T., et al. (2018). An alternative radiolytic route for synthesizing conducting polymers in an organic solvent. *New J. Chem.* 42, 8704–8716. doi:10.1039/C8NJ01041B.

Funda, S., Ohki, T., Liu, Q., Hossain, J., Ishimaru, Y., Ueno, K., et al. (2016). Correlation between the fine structure of spin-coated PEDOT:PSS and the photovoltaic performance of organic/crystalline-silicon heterojunction solar cells. *J. Appl. Phys.* 120, 033103. doi:10.1063/1.4958845.

Garreau, S., Duvail, J. L., and Louarn, G. (2001a). Spectroelectrochemical studies of poly(3,4-ethylenedioxythiophene) in aqueous medium. *Synth. Met.* 125, 325–329. doi:10.1016/S0379-6779(01)00397-6.

Garreau, S., Duvail, J. L., and Louarn, G. (2001b). Spectroelectrochemical studies of poly(3,4-ethylenedioxythiophene) in aqueous medium. *Synth. Met.* 125, 325–329. doi:10.1016/S0379-6779(01)00397-6.

Im, S., Park, C., Cho, W., Kim, J., Jeong, M., Kim, J., et al. (2019). Synthesis of Solution-Stable PEDOT-Coated Sulfonated Polystyrene Copolymer PEDOT:P(SS-co-St) Particles for All-Organic NIR-Shielding Films. *Coatings* 9, 151. doi:10.3390/coatings9030151.

Kalantari Fotooh, F., Nateghi, M. R., and Mohammadi, M. (2018). Structural and Electronic Properties of Novel π-Conjugated Aniline-based Oligomers: A Computational Study. *Phys. Chem. Res.* 6, 583–598. doi:10.22036/PCR.2018.119110.1470.

Kamran, M., Ullah, H., Shah, A.-H. A., Bilal, S., Tahir, A. A., and Ayub, K. (2015). Combined experimental and theoretical study of poly(aniline-co-pyrrole) oligomer. *Polymer (Guildf).* 72, 30–39. doi:10.1016/J.POLYMER.2015.07.003.

Kępas, A., Grzeszczuk, M., Kvarnström, C., Lindfors, T., and Ivaska, A. (2007). Polish journal of chemistry. *Pol. J. Chem.* Vol. 81, 2207–2214. Available at: http://yadda.icm.edu.pl/yadda/element/bwmeta1.element.baztech-article-BUJ6-0023-0093 [Accessed May 31, 2019].

Kim, J., Park, C., Im, S., Lee, H., and Kim, J. H. (2019). Effect of molecular weight distribution of PSSA on electrical conductivity of PEDOT:PSS. *RSC Adv.* 9, 4028–4034. doi:10.1039/C8RA09919G.

Mostafaei, A., and Zolriasatein, A. (2012). Synthesis and characterization of conducting polyaniline nanocomposites containing ZnO nanorods. *Prog. Nat. Sci. Mater. Int.* 22, 273–280. doi:10.1016/j.pnsc.2012.07.002.

Roy, S., Fortier, J. M., Nagarajan, R., Tripathy, S., Kumar, J., Samuelson, L. A., et al. (2002). Biomimetic synthesis of a water soluble conducting molecular complex of polyaniline and lignosulfonate. *Biomacromolecules* 3, 937–941. doi:10.1021/bm0255138.

Rubio, M., Orti, E., Pou-Amérigo, R., and Merchán, M. (2001). Electronic Spectra of 2,2‘-Bithiophene and 2,2‘:5‘,2‘ ‘-Terthiophene Radical Cations:  A Theoretical Analysis. *J. Phys. Chem.* 105, 9788–9794. doi:10.1021/JP011824L.

Santos, M. J. L., Brolo, A. G., and Girotto, E. M. (2007). Study of polaron and bipolaron states in polypyrrole by in situ Raman spectroelectrochemistry. *Electrochim. Acta* 52, 6141–6145. doi:10.1016/J.ELECTACTA.2007.03.070.

Tierrablanca, E., Romero-García, J., Roman, P., and Cruz-Silva, R. (2010). Biomimetic polymerization of aniline using hematin supported on halloysite nanotubes. *Appl. Catal. A Gen.* 381, 267–273. doi:10.1016/j.apcata.2010.04.021.

Trchová, M., and Stejskal, J. (2011). Polyaniline: The infrared spectroscopy of conducting polymer nanotubes (IUPAC Technical Report). *Pure Appl. Chem.* 83, 1803–1817. doi:10.1351/PAC-REP-10-02-01.

Wang, X., Ge, M., and Feng, G. (2015). The effects of DMSO on structure and properties of PVA/PEDOT:PSS blended fiber. *Fibers Polym.* 16, 2578–2585. doi:10.1007/s12221-015-5616-z.

Wang, X., Kyaw, A. K. K., Yin, C., Wang, F., Zhu, Q., Tang, T., et al. (2018). Enhancement of thermoelectric performance of PEDOT:PSS films by post-treatment with a superacid. *RSC Adv.* 8, 18334–18340. doi:10.1039/C8RA02058B.

Xia, Y., MacDiarmid, A. G., and Epstein, A. J. (1994). Camphorsulfonic Acid Fully Doped Polyaniline Emeraldine Salt: In situ Observation of Electronic and Conformational Changes Induced by Organic Vapors by an Ultraviolet/Visible/Near-Infrared Spectroscopic Method. *Macromolecules* 27, 7212–7214. doi:10.1021/ma00102a033.

Zhang, K., Zhang, L. L., Zhao, X. S., and Wu, J. (2010). Graphene/polyaniline nanofiber composites as supercapacitor electrodes. *Chem. Mater.* 22, 1392–1401. doi:10.1021/cm902876u.

Zhekova, H., Tadjer, A., Ivanova, A., Petrova, J., and Gospodinova, N. (2007). Theoretical study of the structure and electronic spectra of fully protonated emeraldine oligomers. *Int. J. Quantum Chem.* 107, 1688–1706. doi:10.1002/qua.21241.

Zhou, J., Li, E. Q., Li, R., Xu, X., Ventura, I. A., Moussawi, A., et al. (2015). Semi-metallic, strong and stretchable wet-spun conjugated polymer microfibers. *J. Mater. Chem. C* 3, 2528–2538. doi:10.1039/C4TC02354D.
