# Supplementary figures and images for: A Comparative Study of Biomimetic Synthesis of EDOT-Pyrrole and EDOT-Aniline Copolymers by Peroxidase-like Catalysts: Towards Tunable Semiconductive Organic Materials
Source: Front Chem. 2022 Jun 29;10:915264. doi: 10.3389/fchem.2022.915264 (PMC9278020; doi:10.3389/fchem.2022.915264)

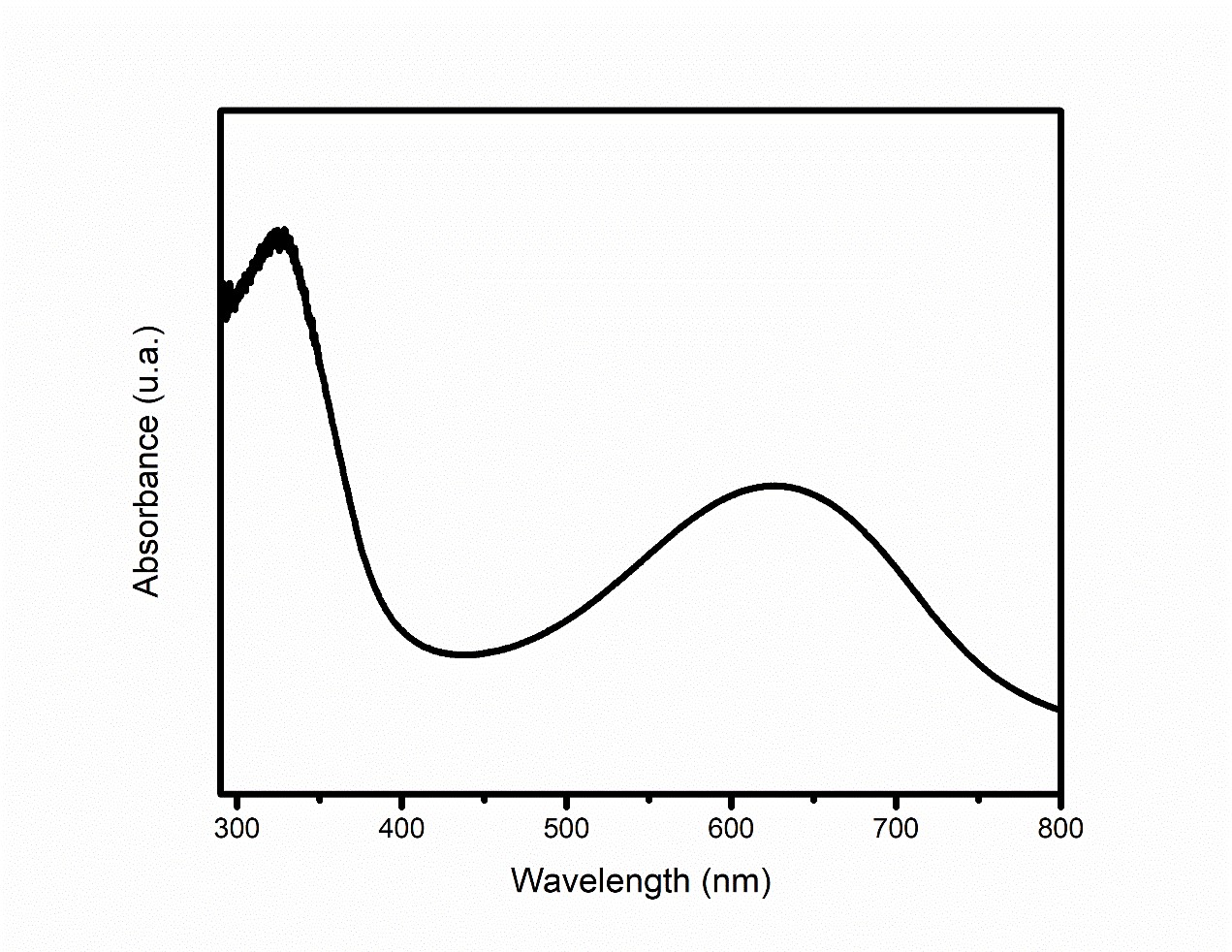

Supplement: Supplementary file 1 [file DataSheet1.ZIP › Figures of SI/Figure S1.jpg]

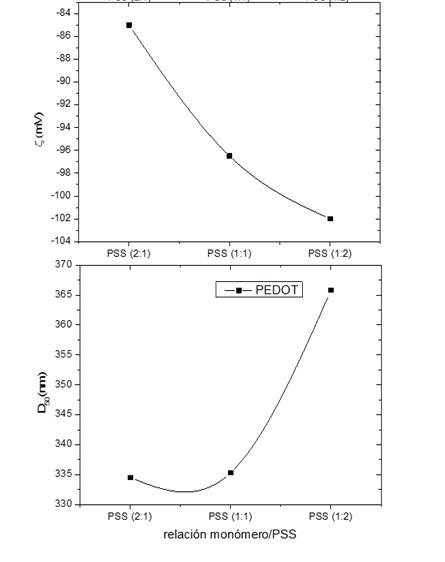

Supplement: Supplementary file 1 [file DataSheet1.ZIP › Figures of SI/Figure S10.jpg]

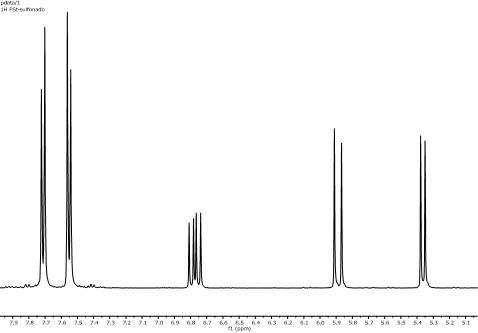

Supplement: Supplementary file 1 [file DataSheet1.ZIP › Figures of SI/Figure S11.jpg]

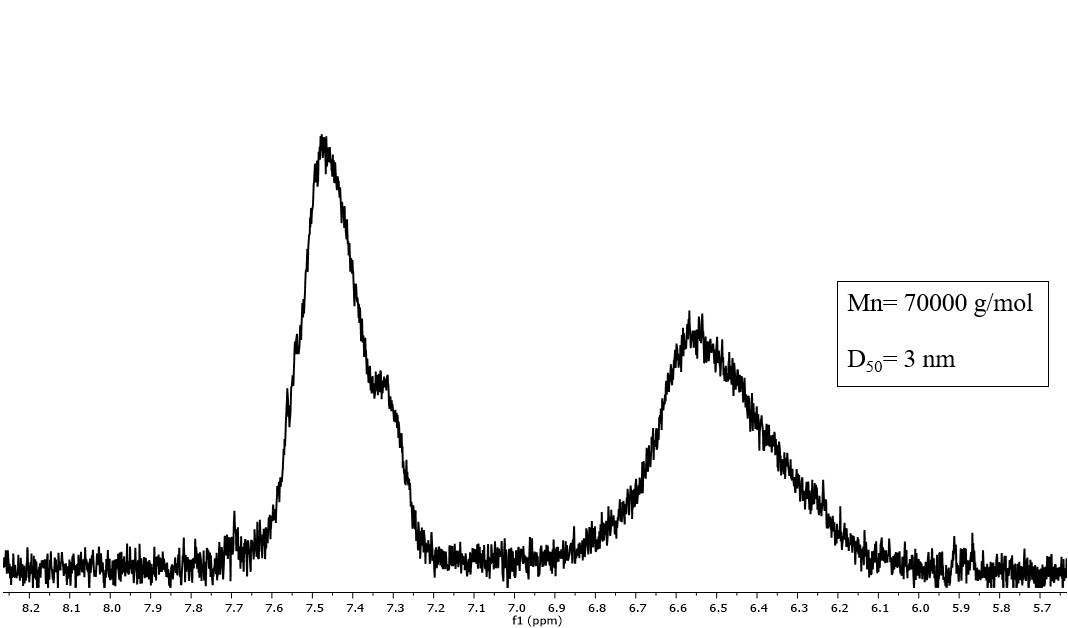

Supplement: Supplementary file 1 [file DataSheet1.ZIP › Figures of SI/Figure S12.jpg]

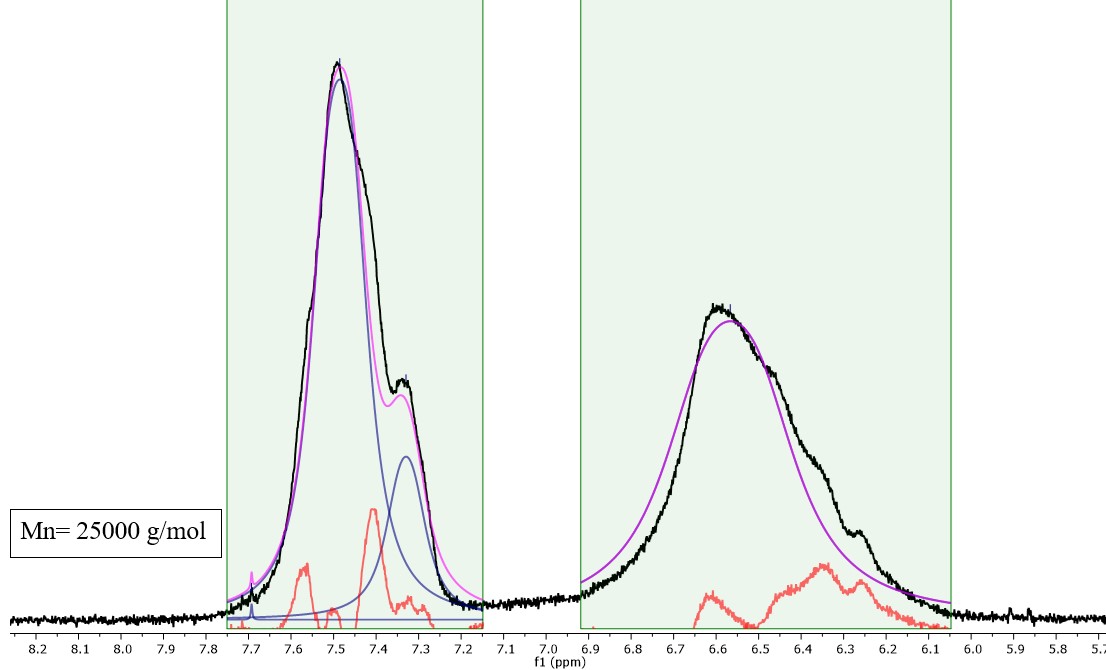

Supplement: Supplementary file 1 [file DataSheet1.ZIP › Figures of SI/Figure S13.jpg]

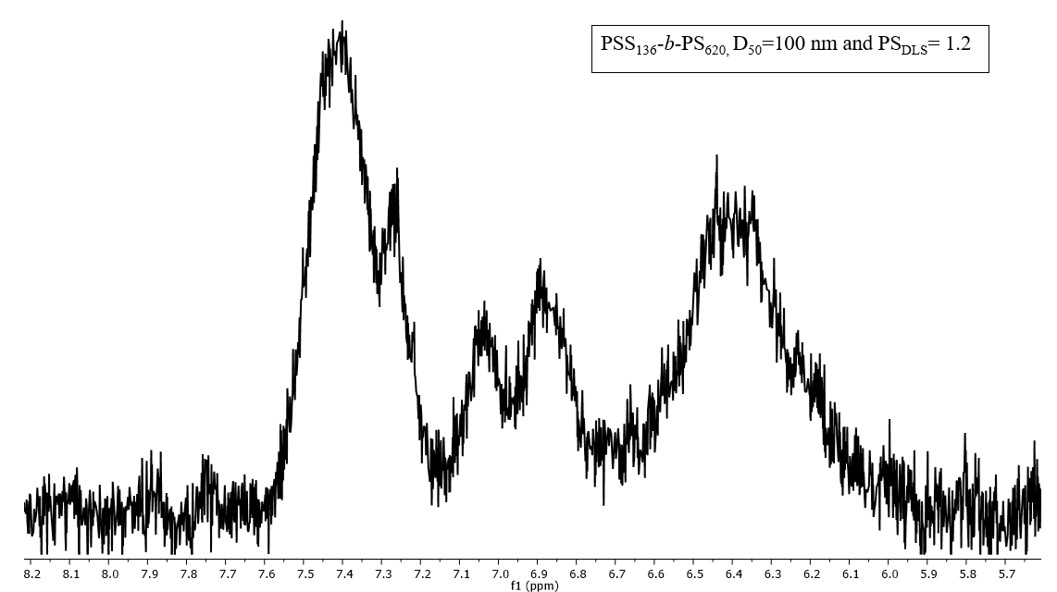

Supplement: Supplementary file 1 [file DataSheet1.ZIP › Figures of SI/Figure S14.jpg]

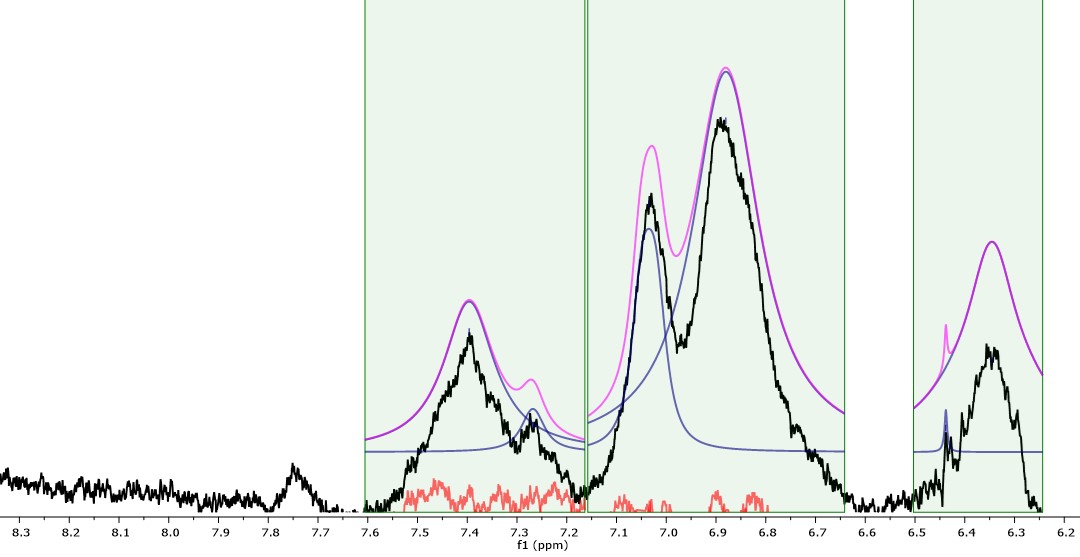

Supplement: Supplementary file 1 [file DataSheet1.ZIP › Figures of SI/Figure S15.jpg]

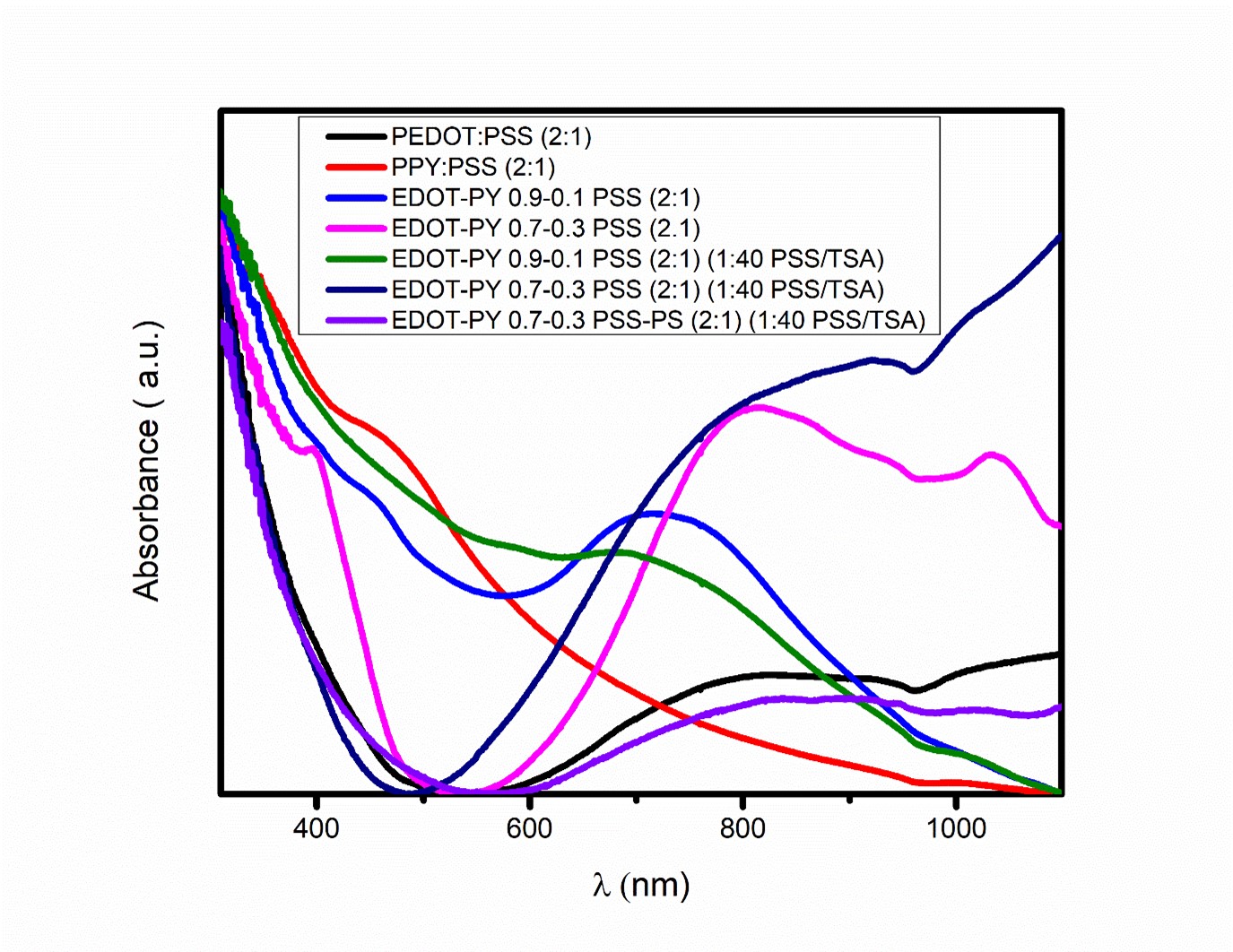

Supplement: Supplementary file 1 [file DataSheet1.ZIP › Figures of SI/Figure S2.jpg]

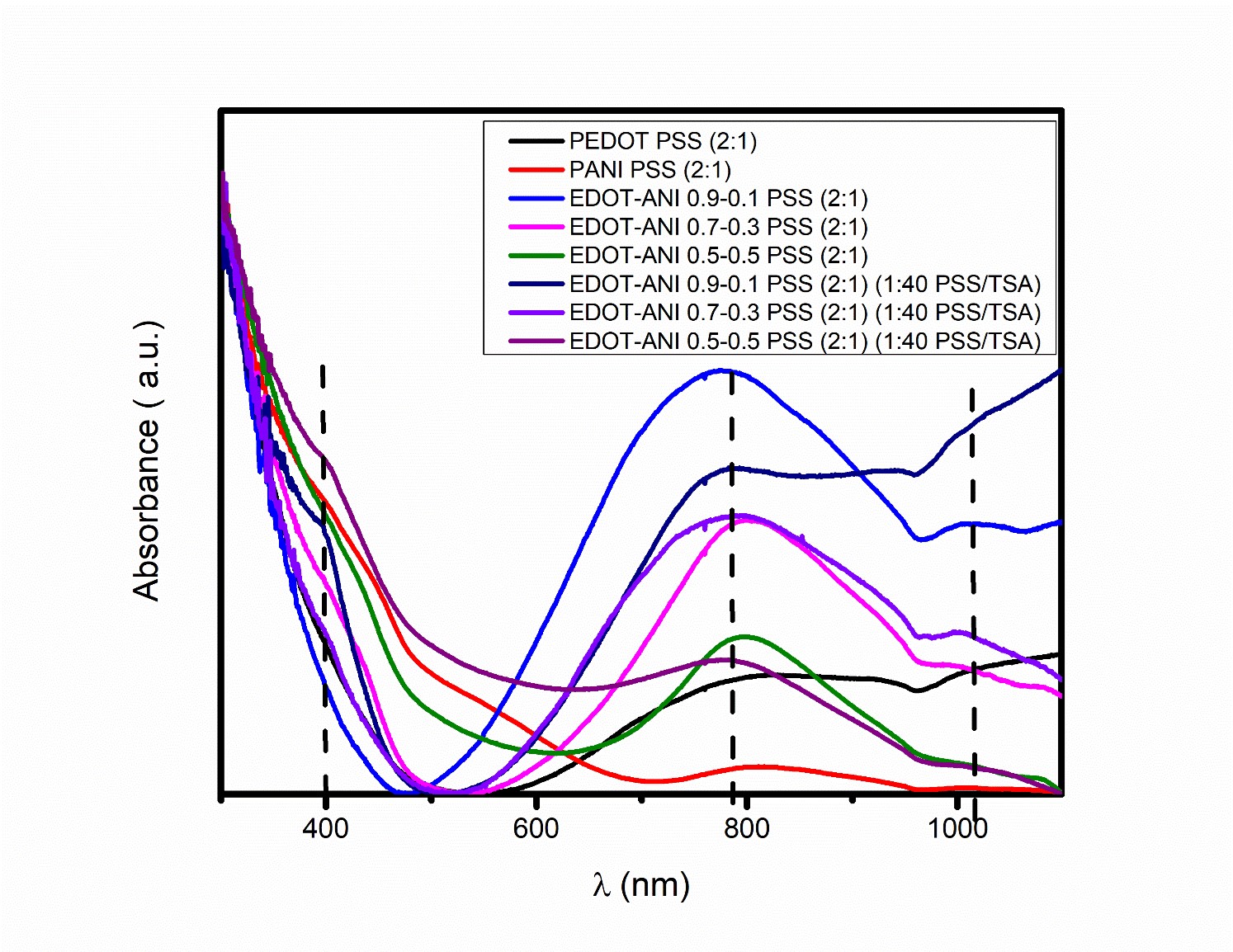

Supplement: Supplementary file 1 [file DataSheet1.ZIP › Figures of SI/Figure S3.jpg]

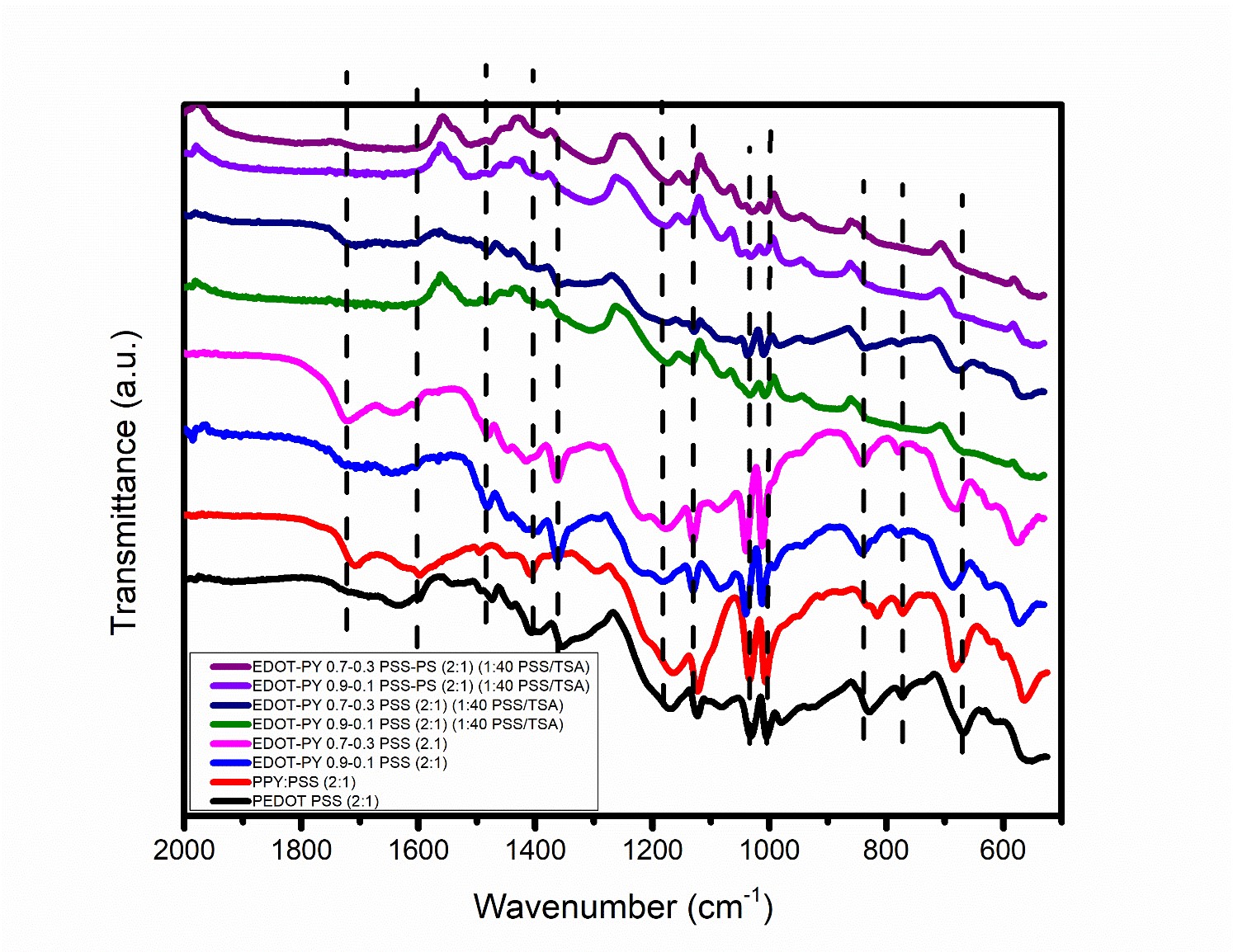

Supplement: Supplementary file 1 [file DataSheet1.ZIP › Figures of SI/Figure S4.jpg]

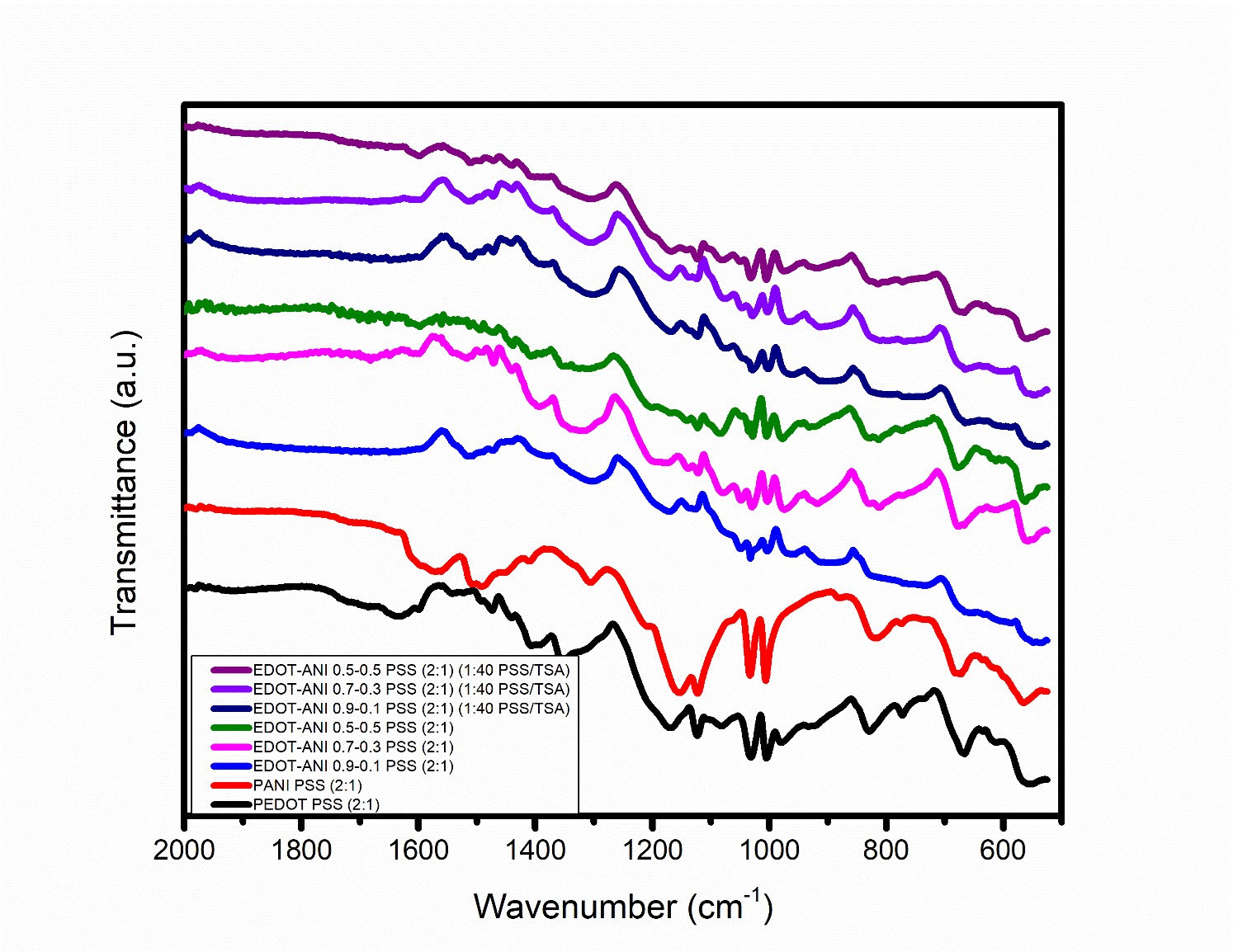

Supplement: Supplementary file 1 [file DataSheet1.ZIP › Figures of SI/Figure S5.jpg]

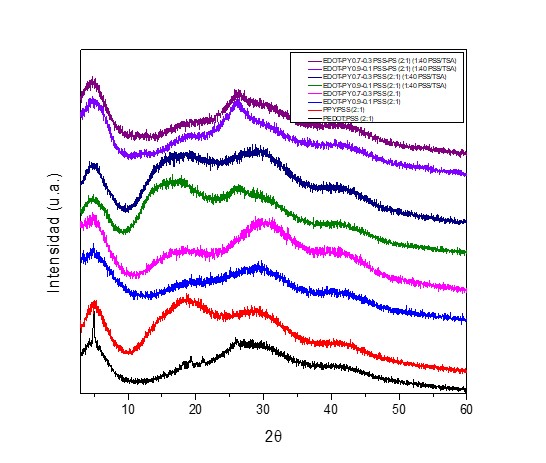

Supplement: Supplementary file 1 [file DataSheet1.ZIP › Figures of SI/Figure S6.jpg]

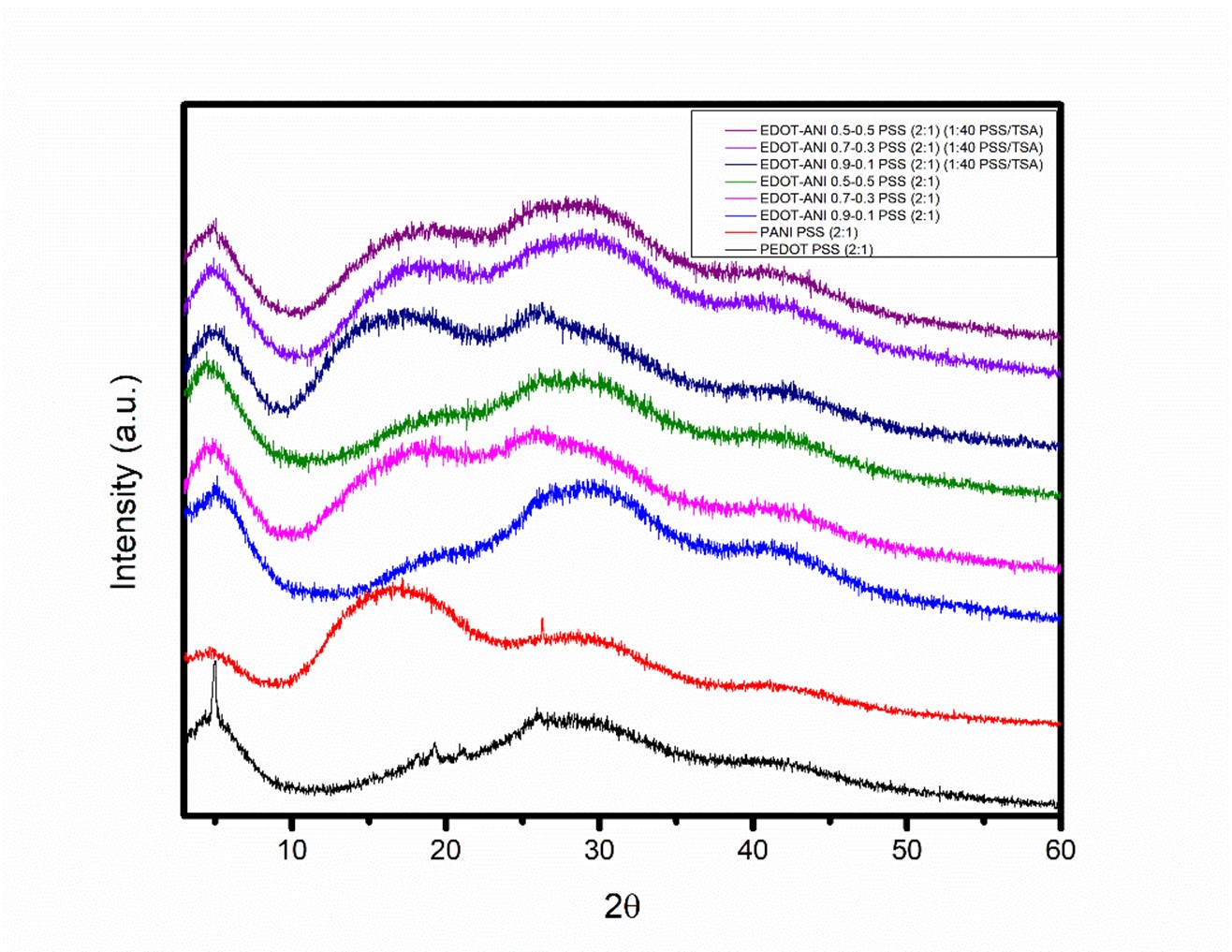

Supplement: Supplementary file 1 [file DataSheet1.ZIP › Figures of SI/Figure S7.jpg]

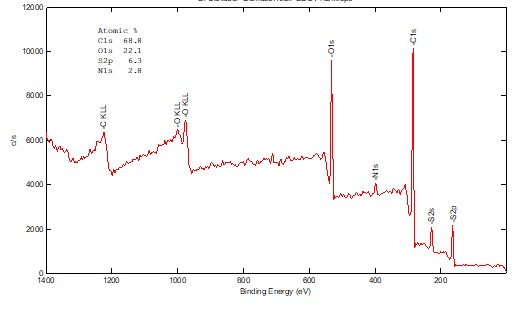

Supplement: Supplementary file 1 [file DataSheet1.ZIP › Figures of SI/Figure S8.jpg]

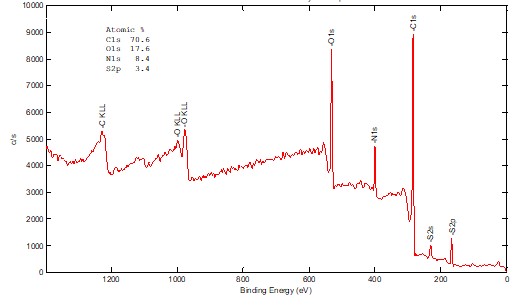

Supplement: Supplementary file 1 [file DataSheet1.ZIP › Figures of SI/Figure S9.jpg]

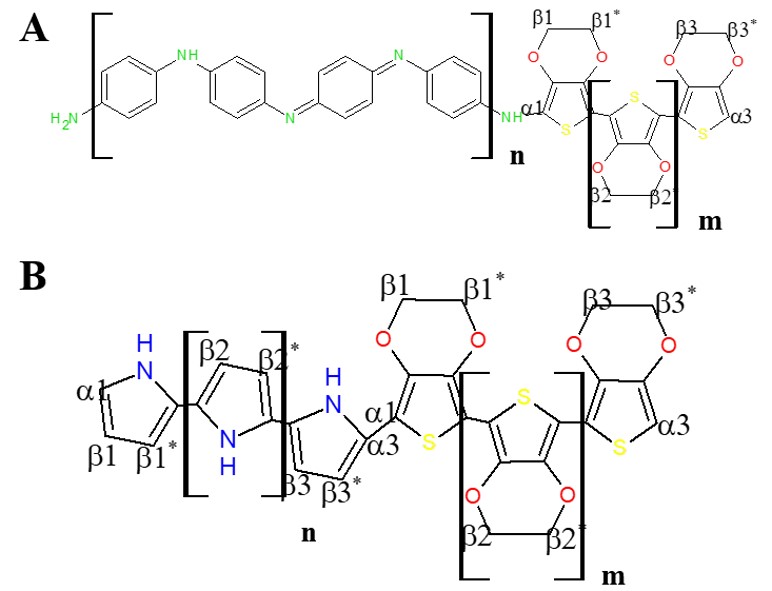

Supplement: Supplementary file 1 [file DataSheet1.ZIP › Figures of SI/Scheme S1.jpg]

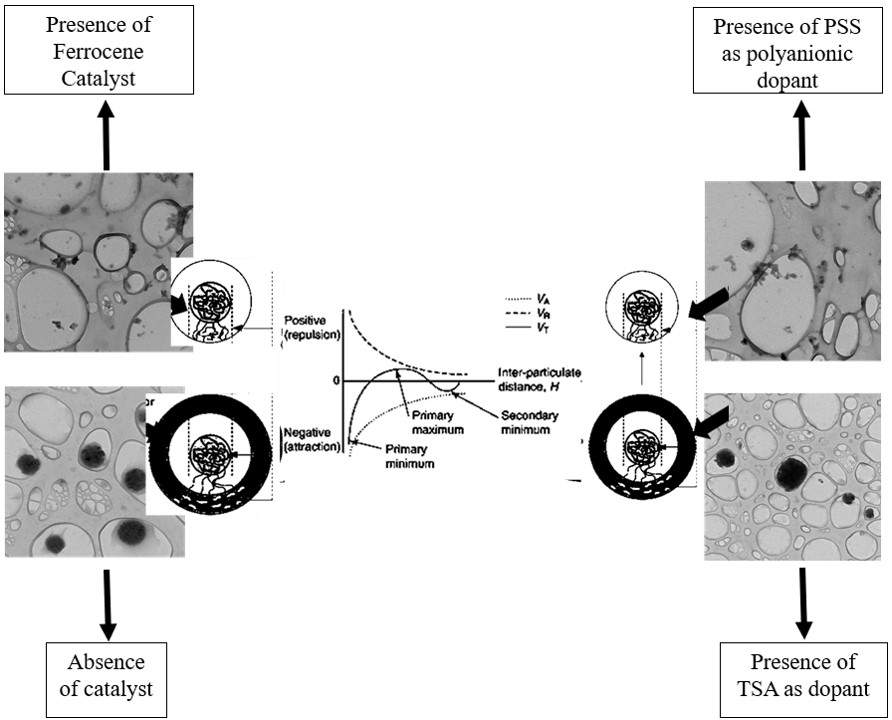

Supplement: Supplementary file 1 [file DataSheet1.ZIP › Figures of SI/Scheme S2.jpg]
